# Optineurin Regulates the Interferon Response in a Cell Cycle-Dependent Manner

**DOI:** 10.1371/journal.ppat.1004877

**Published:** 2015-04-29

**Authors:** Pierre Génin, Frédérique Cuvelier, Sandrine Lambin, Josina Côrte-Real Filipe, Elodie Autrusseau, Christine Laurent, Emmanuel Laplantine, Robert Weil

**Affiliations:** 1 Laboratoire de Signalisation et Pathogenèse, CNRS UMR3691, Institut Pasteur, Paris, France; 2 Plate-Forme Protéomique, Institut Pasteur, Paris, France; University of Pittsburgh, UNITED STATES

## Abstract

Viral invasion into a host is initially recognized by the innate immune system, mainly through activation of the intracellular cytosolic signaling pathway and coordinated activation of interferon regulatory factor 3 (IRF3) and nuclear factor kappa B (NF-κB) transcription factors that promote type I interferon gene induction. The TANK-binding Kinase 1 (TBK1) phosphorylates and activates IRF3. Here, we show that Optineurin (Optn) dampens the antiviral innate immune response by targeting the deubiquitinating enzyme CYLD to TBK1 in order to inhibit its enzymatic activity. Importantly, we found that this regulatory mechanism is abolished at the G2/M phase as a consequence of the nuclear translocation of CYLD and Optn. As a result, we observed, at this cell division stage, an increased activity and phosphorylation of TBK1 that lead to its relocalization to mitochondria and to enhanced interferon production, suggesting that this process, which relies on Optn function, might be of major importance to mount a preventive antiviral response during mitosis.

## Author Summary

The innate immune system has evolved to detect and neutralize viral invasion. Triggering of this defense mechanism relies on the production and secretion of soluble factors that stimulate an intracellular antiviral defense mechanism. The protein Optineurin was shown to negatively regulate this process. Importantly, we discovered the mechanism by which Optineurin inhibits antiviral activity and showed that this regulation is prevented during a critical step of cell division leading to enhancement of the cellular defense mechanism. This paper shows that the antiviral immune system is controlled during the cell cycle and that Optineurin-mediated induction of this system might serve to protect cells from infection during cell division.

## Introduction

Innate immunity is a host mechanism found in most multicellular organisms that serves as a first line of defense against microbial pathogens. The innate immune response results in the production of immune modulatory cytokines and the mobilization of innate immune cells. Detection of pathogen associated molecular patterns (PAMPS) by the pattern-recognition receptors (PRR) activates intracellular signaling pathways that culminate in the production and secretion of pro-inflammatory cytokines, chemokines and type I IFN, i.e. IFN-α and IFN-β. Once secreted, these cytokines stimulate transcription of IFN-stimulated genes (ISGs), products of which prevent virus spreading and activate the adaptive immune responses [1,2].

Among these PRRs, membrane-bound Toll-like receptors (TLRs) sense nucleic acids from microbial genome, bacterial lipopolysaccharides or viral coat proteins, while early RNA replicative intermediates are mainly detected by retinoic acid inducible-I (RIG-I)-like receptors (RLRs) including cytosolic RNA helicases RIG-I and Mda-5 (melanoma differentiation-associated gene 5) (reviewed by [3]). In addition, cytosolic DNA-dependent RNA polymerase III can convert AT-rich double stranded DNA into dsRNA that can be subsequently sensed by RIG-I [4]. Recognition of double-stranded viral RNAs bearing 5’-triphosphate by RIG-I allows its interaction with the mitochondrial adaptor protein MAVS, also known as Cardif/IPS-1/VISA [5,6]. Engagement of MAVS, localized at the outer mitochondrial membrane, leads to the assembly of a signaling platform and to the activation of interferon regulatory (IRFs) and nuclear factor-κB (NF-κB) transcription factors, which cooperatively activate type I IFN gene transcription [7]. In contrast to NF-κB activation that relies on the degradation of cytoplasmic inhibitors, activation of IRF3 and IRF7 in the cytoplasm occurs directly through their phosphorylation by the TANK-binding kinase-1 (TBK1) and IKKε kinases that present sequential and structural homologies with the IκB kinases, IKKα and IKKβ [8]. These phosphorylations induce conformational changes in IRF3 that promote its dimerization, nuclear transport, and association with co-activators such as CBP/p300 and PCAF to stimulate their transcriptional activities [9,10].

TBK1 is a serine/threonine kinase functioning as a key node protein in several cell signaling pathways including innate immune response, autophagy-mediated elimination of bacteria and, under physiological conditions, cell growth and proliferation [11–15]. TBK1 is composed of a kinase domain, an ubiquitin-like (UBL) domain, a dimerization domain and a C-terminal adaptor-binding motif [16]. TBK1 is regulated by phosphorylation on Serine 172 (S172) within the classical kinase activation loop. The upstream kinase activating TBK1 in response to viral infection is not yet known, although genetic and pharmacological studies suggested that TBK1 could be activated by IKKβ, as well as by autophosphorylation that can be facilitated by Glycogen Synthase Kinase (GSK)-3β interaction [17,18]. Several phosphatases have been identified as regulators of TBK1 phosphorylation, including the inositol 5’ phosphatase SHIP-1 or protein phosphatase Mg^2+/^Mn^2+^ dependent 1B (PPM1B/PP2Cβ), during TLR3 stimulation or virus infection, respectively [19,20]. TBK1 K63-linked polyubiquitination was recently shown to be important for LPS- and RLR-induced IFN production. In response to RNA virus infection, the E3 ligases Mind Bomb 1 and 2 (MIB1 and 2) were shown to couple K63-linked ubiquitin to TBK1 on residues K69, K154 and K372 [12,16], while its ubiquitination is mediated by Ndrp1 in LPS-stimulated cells [21]. At the opposite, several deubiquitinases cleave K63-linked polyubiquitination to terminate TBK1-mediated pathway, including deubiquitinating enzyme cylindromatosis (CYLD), ubiquitin-editing enzyme A20 (also known as TNFAIP3) and Ubiquitin-Specific Protease 2b (USP2b) [22–25]. Recently, the RING finger protein 11 (RNF11) was reported to functionally cooperate with TAX1BP1 to inhibit K63-linked polyubiquitination of TBK1 and IFN production and to remove K63-linked chains from this kinase [26]. Recent observations have demonstrated that NEMO (NF-κB essential modulator) bridges the NF-κB and IRF signaling pathways: MIB-dependent attachment of K63-linked chains to TBK1 following viral RNA recognition induces its interaction with NEMO and the recruitment of this complex to MAVS [16], while linear ubiquitination of NEMO dissociates the MAVS-TRAF3 complex to inhibit the RIG-I signaling for the benefit of the NF-κB-dependent pathway [27].

Optineurin (tineurin (TE.DATA the MAVS-TRAOptn), also called NRP (NEMO-related protein) or FIP-2 (adenovirus E3-14.7K-interacting protein) exhibits 53% of sequence similarities with NEMO [28,29]. Despite these similarities, Optn does not play a critical role in the regulation of the NF-κB pathway except in some circumstances [29–31]. Optn has been linked to different pathologies since mutations in the Optn gene have been associated with different primary and juvenile forms of Open-Angle Glaucoma (the most frequent mutation being E50K) as well as with Amyotrophic Lateral Sclerosis (ALS) and polymorphisms of Optn gene are risk factor for Paget’s disease of bone [32]. So far, Optn has been involved in at least four apparently unrelated functions: membrane trafficking, antiviral innate immunity, autophagy and regulation of mitosis (reviewed in [33]). The function of Optn in the TBK1-dependent pathways was suggested by different observations. A link between Optn and the innate immune response has been first suggested by its interaction with TBK1 [34] and its identification as a substrate of this kinase in response to LPS stimulation [14,35]. Second, using RNAi and overexpression experiments, Mankouri et al. [36] suggested that Optn has an inhibitory effect on the transcriptional activation of IFN-B gene in response to virus infection. In addition, ALS-associated mutants of Optn abrogate the inhibition of IRF3 activation in response to MDA5 or TRIF overexpression [37]. However, the negative regulatory function of Optn in the TBK1-dependent pathways remains controversial. Thus, using knocked-in mice expressing an Ub-binding defective mutant of Optn (Optn-D477N), Gleason et al. [35] concluded that Optn enhanced TBK1 activation in response to LPS or dsRNA based on the finding that TBK1/IRF3/IFN-B signaling pathway is impaired in bone marrow-derived macrophages obtained from Optn-D477N knock-in mice compared to wild-type mice. Similarly, it was found that the Ub-binding function of Optn was necessary for optimal TBK1 and IRF3 activation and that macrophages and dendritic cells from knocked-in mice expressing an Ub-binding defective mutant of Optn (Optn-470T) had diminished IFN-β production upon LPS stimulation [30].

In the present study, we confirmed that Optn plays a negative regulator role on the virus-induced IFN pathway by targeting TBK1 activity [36,37]. We further demonstrated that Optn-mediated recruitment of the deubiquitinase CYLD to TBK1 is responsible for this inhibitory effect. We had previously shown that Optn negatively regulates the activity of the mitotic kinase Polo-like 1 (Plk1) [38]. During the G2/M phase, Optn is phosphorylated on Serine 177 by Plk1 and subsequently undergoes nuclear translocation, to control Plk1 activity for proper mitotic progression through a feedback loop mechanism. Interestingly, our results indicate that the TBK1/Optn/CYLD complex is disrupted during G2/M phase as a consequence of Optn and CYLD accumulation to the nucleus, leading to enhancement of TBK1 activity and relocalization of its active form to the mitochondria. Release of the CYLD-mediated inhibition of TBK1 during this phase results in enhanced IFN/ISG signaling pathway, independently of viral infection. Overall, our data demonstrate that Optn dampens the IFN signaling pathway by targeting TBK1 activity and that this process is abolished during the G2/M phase, resulting in enhanced TBK1/IRF/IFN activity and consequently in augmented antiviral immune response.

## Results

### Optn acts as a negative regulator of the antiviral innate immune response

To assess the role of Optn in antiviral host response, we used HeLa cell clones depleted of Optn using stably introduced shRNA, and stably complemented with either wild-type (wt) or mutated shRNA-resistant Optn plasmids that we previously generated to ensure a uniform ectopic expression equivalent to the endogenous level of Optn [38]. As previously observed by Mankouri et al. [36], infection of Optn-deficient cells with Sendai Virus (SeV) led to increased IFN-B gene expression (2- to 4-fold) compared to control or to wt Optn reconstituted cells (S1A Fig). Enhancement of the IFN-Stimulated Gene transcription such as ISG15 and Viperin, was also observed in Optn-deficient cells at 9h post-infection, compared to control or Optn-reconstituted cells, without affecting the NF-κB-dependent transcription of IκBα gene (S1B Fig). Consistent with this Optn-dependent regulation of the IFN response, we observed a 2-fold increase in the IFN-β protein levels secreted from poly(I:C)-stimulated Optn-deficient cells (S1C Fig). Altogether, these results are consistent with a specific effect of Optn on the IRF3/7-mediated activation of the IFN pathway. We further observed that transfection of poly(I:C) led to higher IFN-B expression in Optn-depleted compared to Optn-reconstituted cells and that increasing amount of transfected Optn-expressing plasmid inhibited poly(I:C)-induced IFN-B gene transcription in a dose-dependent manner (S1D and S1E Fig).

### Ubiquitin-binding activity and phosphorylation of Optn are required for its inhibitory function in innate immunity

**Fig 1:**
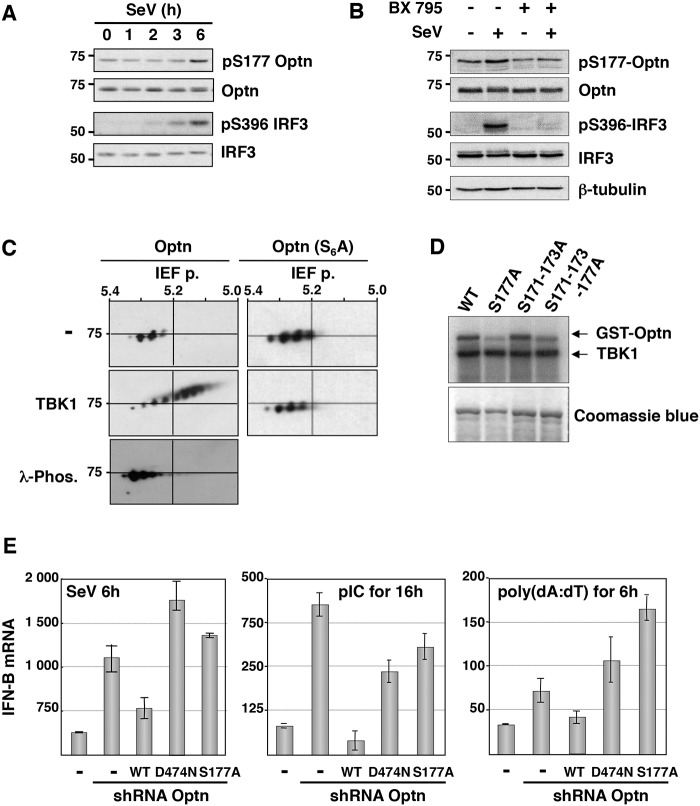
Optn negative effect on the virus-induced interferon pathway requires its phosphorylation and ubiquitin-binding activity. (A) Phosphorylation status of Optn (on S177) and IRF3 (on S396) were determined by Western blot in uninfected HeLa cells and at different time after infection by Sendai virus. (B) Total cell lysates from HEK293T infected for 6h with SeV in the presence or absence of the TBK1-specific inhibitor BX795 were analyzed by Western blot using anti-pS177 Optn, anti-Optn, anti-pS396 IRF3, anti-IRF3 and anti-tubulin antibodies. (C) Total cell lysates extracted from HEK293T cells expressing either VSV-Optn (Optn, left panels) or VSV-Optn S162-170-171-173-174-177A (Optn-S_6_A, right panels) were transfected with empty vector (-) or with TBK1-expressing plasmid (TBK1) and subjected to two-dimensional gel electrophoresis. Optn isoforms were analyzed by immunoblotting with an anti-VSV antiserum. As control, lysates were pre-treated with lambda phosphatase (λ-Phos.) at 30°C. (D) HEK293T cells were transiently transfected with Flag-TBK1. TBK1 kinase assays were carried out using Flag immunoprecipitates as enzyme and GST-Optn wt, GST-Optn S177A, GST-Optn S171-173A or GST-Optn S171-173-177A as substrates. (E) IFN-B mRNA levels were determined by RT-QPCR in control HeLa cells, Optn-deficient cells and deficient cells reconstituted with wt, D474N- or S177A-mutated forms of Optn following infection by SeV or stimulation with dsRNA (pIC) or dsDNA (poly(dA):(dT)) and presented as mRNA transcripts levels related to the mRNA of 18S (set at 100). Mean ± SD values of expression levels are shown.

Since it was previously reported that TBK1 phosphorylates Optn on Serine 177 (S177) in response to LPS [14,35], we determined the S177 phosphorylation status of Optn in SeV-infected cells by Western blot analysis using a phospho-specific antibody (pS177) (S2A Fig, [38]). We observed basal phosphorylation of Optn S177 that was enhanced in response to virus infection, with a kinetic similar to that of IRF3 (on Serine 396) that reflects its activation (Fig 1A). As for IRF3 phosphorylation, virus-induced phosphorylation of Optn at S177 was abrogated in the presence of TBK1 inhibitor BX795 (Fig 1B). Consistent with the presence of several consensus phosphorylation sites for TBK1 in the insert region of Optn (see S2A Fig), multiple phosphorylated forms of Optn were separated in two-dimensional gel electrophoresis when VSV-tagged Optn was co-expressed with TBK1 in HEK293T cells (Fig 1C). These post-translational modified forms disappeared after treatment with λ-phosphatase or after substitutions of the Serine residues present in the consensus motif for TBK1 by Alanine residues. Kinase assays performed using immunoprecipitated TBK1 (as source of kinase) and mutants of Optn on multiple Serine (as substrates) further indicated that S177 was the preferential site of phosphorylation by TBK1 (Fig 1D). The inhibitory effect of wt Optn on virus-induced IFN-B gene expression was abolished by the S177A substitution and by mutation in its ubiquitin-binding domain (D474N), but was not affected by the pathogenic E50K mutation (Figs 1E, left panel and S2B–S2C). Similar results were obtained when HeLa cells were transfected with dsRNA or dsDNA (Figs 1E, middle and right panels, respectively and S2B). These data indicated that the inhibitory function of Optn on stimulated IFN-B expression requires integrity of its ubiquitin-binding domain (consistent with previous results [36]) and of its main phosphorylation site.

### Optn inhibitory effect targets the TBK1 kinase activity

**Fig 2:**
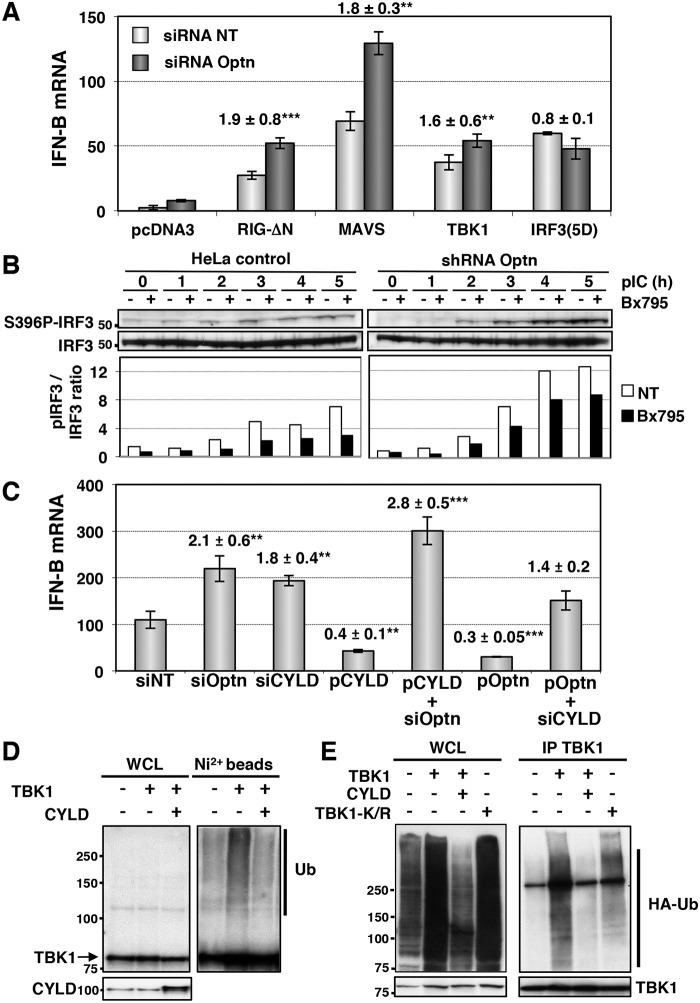
Optn inhibitory effect requires CYLD and targets TBK1 ubiquitination. (A) IFN-B mRNA levels were determined in HeLa cells cotransfected with non-targeting or Optn-specific siRNAs together with empty vector or plasmid expressing either constitutively active form of RIG (RIG-ΔN) and IRF3 (IRF3-5D) or native form of MAVS and TBK1 and presented as in Fig 1E. Mean ± SD values of induction folds (corresponding to the ratio of the IFN-B expression level observed in Optn-deficient cells to that observed in control cells) are shown. ** p values < 0.01, *** p values < 0.001. (B) Cell lysates from control and stable Optn-depleted cells transfected with poly(I):poly(C) (pIC) for the indicated periods of time (h), in the absence or in the presence of the TBK1 inhibitor BX795, were immunoblotted with anti-pS396 IRF3 and IRF3 antibodies. Quantification of the signals obtained is presented as a ratio of phosphorylated levels versus total protein levels in the graph below. (C) IFN-B mRNA levels determined by RT-QPCR in HeLa cells transfected with non-targeting (siNT), Optn- or CYLD-specific siRNAs or/and with plasmids expressing CYLD or Optn and then stimulated by poly(I):poly(C) are presented as in Fig 1E. Mean ± SD values of induction folds (corresponding to the ratio of the IFN-B expression levels relative to that observed in cells transfected with non-targeting siRNA) are shown. ** p values < 0.01, *** p values < 0.001. (D) Nickel-Sepharose-purified ubiquitinated products (Ni^2+^ beads) and whole cell lysates (WCL, 5% of the total lysates) were resolved on SDS-PAGE and analyzed by Western blot using anti-TBK1 and anti-CYLD antibodies. The molecular weights (kDa) are represented on the left of each immunoblot. (E) TBK1-containing complexes were immunoprecipitated from pIC-stimulated HeLa cells overexpressing HA-tagged ubiquitin and wt TBK1 alone and together with and CYLD or with TBK1 mutated form mutated on the ubiquitination sites K30 and K401 (TBK1-K/R). TBK1-isolated complexes were resolved on SDS-PAGE and analyzed by Western blot using anti-HA and anti-TBK1 (control) antibodies. The molecular weights (kDa) are represented on the left of each immunoblot.

To identify the step of the IFN pathway targeted by the inhibitory function of Optn, we overexpressed native or constitutively active form of components of the RIG/IRF/IFN signaling pathway in Optn-depleted cells (Figs 2A and S2D). Transfection of plasmids encoding the truncated form of RIG-I (RIG-ΔN), the native forms of MAVS and TBK1 resulted in a 1.6- to 1.9-fold enhancement of the IFN-B gene expression in cells co-transfected with Optn-specific siRNAs compared to those transfected with non-target siRNAs, while no effect was observed after transfection of the constitutive form of IRF3 (IRF3-5D). These results strongly suggested that Optn inhibitory effect targets TBK1 kinase activity, this hypothesis being supported by the increased S172 TBK1 phosphorylation (maximum of 1.8-fold) observed in Optn-deficient cells (S2E Fig). Depletion of Optn also led to an up to 3-fold enhancement of IRF3 S396 phosphorylation in response to dsRNA that was reduced by pretreatment of the cells with the TBK1 specific inhibitor (BX795), arguing again for an effect of Optn on TBK1 (Fig 2B).

Optn has been identified as a receptor for bacterial-induced autophagy and its phosphorylation on S177 by TBK1 was shown to control its interaction with LC3 [14]. This observation suggests that Optn could inhibit the innate immune response by inducing the autophagy-mediated degradation of TBK1. We further tested this hypothesis and found that induction of autophagy by rapamycin or serum deprivation (S3A Fig, lanes 2 and 3) or inhibition of autophagy by bafilomycin (S3A Fig, lanes 4–6) did not affect TBK1 protein levels, nor altered the stimulated IFN-B gene expression in wild-type or Optn-depleted cells (S3C–S3E Fig). Furthermore, no inhibition or enhancement was observed using 3-Methyladenine (3-MA), that blocks autophagosome formation via the inhibition of type III Phosphatidylinositol 3-kinases (S3D Fig), as confirmed by inhibition of the S6 Ribosomal protein phosphorylation (S3E Fig, [39]). This observation argued against a potential effect of sequestration of Optn-containing complexes into autophagosomes on the IFN-B expression. These results were consistent with the observation that disruption of the Optn-LC3 interaction (mutant F178R, described in [14]) did not affect the inhibitory effect of Optn on the pIC-stimulated IFN-B gene expression (S3B Fig).

Taken together, these results indicated that the inhibitory effect of Optn on the IFN-B gene expression was not related to Optn function in the autophagic degradation pathway. Since TBK1 activity is regulated by its phosphorylation and ubiquitination [40], we hypothesized that Optn could exert its inhibitory effect on TBK1 activity by recruiting and/or activating a phosphatase or a deubiquitinase (see below) that, in turn, could inactivate TBK1. Interaction of Optn with the myosin phosphatase complex MYPT1/PP1β has been shown to exert a negative regulatory effect on Plk1 phosphorylation during the G2/M phase [38], suggesting that this complex could inhibit TBK1 activity by dephosphorylation. However, expression of an Optn mutant that is defective for its association with MYPT1 (Optn L150P/L157P, [38]) did not significantly affect the IFN-B gene expression stimulated with poly(I:C) (see S2B Fig). These results suggested that interaction of Optn with the MYPT1/PP1β phosphatase complex could not account for the inhibitory effect of Optn on TBK1 activity, thus suggesting that either another phosphatase or a deubiquitinase could mediate this inhibitory function.

### CYLD affects dsRNA-induced IFN-B gene transcription in an Optn-dependent manner

Since the E3 ligases Mind Bomb 1 and 2 (MIB1 and MIB2) have been recently shown to promote the attachment of K63-linked ubiquitin chains to TBK1 following virus infection [12] and that this modification is required for TBK1-induced gene expression and kinase activation [40], we hypothesized that Optn may act as an inhibitor of the antiviral signaling pathway by targeting a deubiquitinase to TBK1. Interestingly, Optn interacts with the tumor suppressor gene CYLD, a deubiquitinase (DUB) that catalyzes the cleavage of linear and K63-linked polyubiquitin chains and with A20, a DUB for K63-linked polyubiquitinated signaling mediators such as TRAF6 and RIP1 [41–44]. To test whether any of these two DUBs could be involved in the Optn-mediated regulation of innate immunity, we performed depletion experiments in dsRNA-stimulated HeLa cells. Strikingly, the shRNA-mediated depletion of CYLD resulted in enhancement of both IFN-B gene expression and NF-κB-mediated transcription, while depletion of A20 affected only the NF-κB-mediated transcription (S4A and S4B Fig). We therefore focused on the role of CYLD in Optn-mediated inhibition of the innate immunity. As shown in Figs 2C and S4C, siRNA-mediated depletion and overexpression of CYLD or Optn led to similar enhancement and inhibition of IFN-B gene expression, respectively. Interestingly, CYLD and Optn were dependent on each other for their inhibitory effect, since CYLD and Optn overexpression could not inhibit IFN-B in the absence of each other (Fig 2C). Similar dependencies between Optn and CYLD was also observed in stable cell lines, since overexpression of CYLD inhibited the poly(I:C)-stimulated IFN-B gene expression in control HeLa cells, but not in cells stably depleted for Optn (S4D Fig). These results clearly indicate that Optn-mediated inhibition of the antiviral immune response is dependent on CYLD. Accordingly, an enzymatically inactive mutant of CYLD (H871N or H/N, [45]) and a mutant of interaction of Optn with CYLD (H486R mutant, [46]) failed to inhibit poly(I:C)-stimulated IFN-B gene expression compared to wt CYLD and Optn, respectively (S4D and S4E Fig). We next isolated ubiquitinated proteins from His-tagged ubiquitin-overexpressing cells using nickel (Ni^2+^) beads under denaturing conditions and monitored the ubiquitination of exogenously expressed TBK1 by Western blot using anti-TBK1 antibodies (Figs 2D and S4F). As expected, expression of CYLD resulted in reduction of the ubiquitination levels of overexpressed TBK1, further indicating that CYLD activity targeted TBK1 (Fig 2D). Similar results were obtained when TBK1 was immunoprecipitated from pIC-stimulated HeLa cells overexpressing wt TBK1 and CYLD (Figs 2E and S4G). Specificity of the TBK1 ubiquitination signal was confirmed by the reduction observed when TBK1 was mutated on the ubiquitination sites K30 and K401 (Fig 2E), as previously described [40]. We therefore concluded that Optn targets the deubiquitinase activity of CYLD to TBK1 to prevent its activity and to dampen the antiviral response.

### Formation of the TBK1/Optn/CYLD complex is disrupted during the G2/M transition

**Fig 3:**
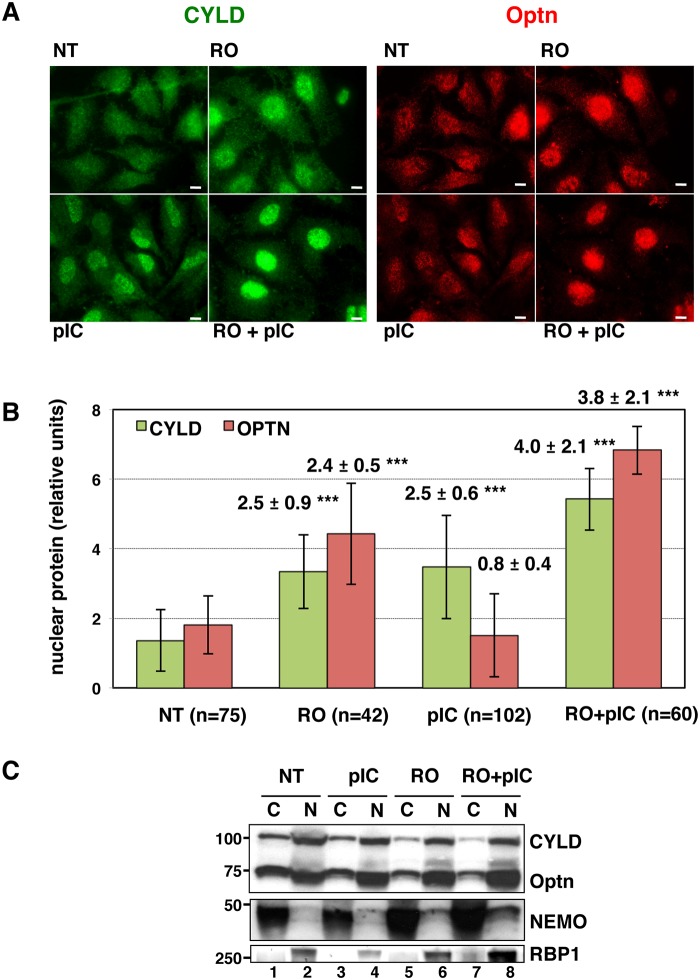
CYLD and Optn accumulate in the nuclei of G2/M synchronized cells. (A) Changes in localization of CYLD and Optn were monitored by immunofluorescence microscopy in HeLa cells untreated (NT) or synchronized in G2/M by RO-3306 (RO, inhibitor of Cdk1 activity) and either stimulated by poly(I):poly(C) (pIC) or left untreated. Bars = 10 μm. (B) Quantification of cells expressing nuclear CYLD or Optn. Fold differences between cell numbers exhibiting nuclear CYLD or Optn relative to the respective non-treated condition (NT) are indicated. *** p values < 0.001. (C) Cytoplasmic (C, lanes 1, 3, 5 and 7) and nuclear extracts (N, lanes 2, 4, 6 and 8) of HeLa cells obtained by subcellular fractionation treated with RO-3306 or left untreated (NT) in the presence or in the absence of pIC were immunoblotted with anti-CYLD, anti-Optn, anti-NEMO or anti-RBP1 (largest subunit of RNA polymerase II) antibodies. Crosscontaminations were assessed by immunoblotting for the cytosolic NEMO protein and nuclear RNA Polymerase II B subunit (RBP1) as compartment specific markers. The molecular weights (kDa) are represented on the left of each immunoblot.

Since we have previously shown that Optn accumulates in the nucleus during the G2/M transition [38], we speculated that this Optn relocation could prevent Optn/CYLD-mediated inhibition of TBK1 activity. Interestingly, we found that not only Optn, but also CYLD accumulated into the nucleus when cells were treated with RO-3306 (a Cdk1 inhibitor that synchronize cells at the a G2/M boundary) and/or dsRNA (Fig 3A and quantification in 3B). CYLD was also detected in the nucleus of cells stimulated with dsRNA alone (Fig 3A and 3B), suggesting that accumulation of CYLD to the nucleus might be also part of the TBK1 activation mechanism after dsRNA stimulation of unsynchronized cells. Subcellular fractionation followed by Western blot analyses confirmed the reduced levels of CYLD and Optn in the cytosolic compartment of RO- and RO/poly(I:C)-treated cells (Fig 3C, compare lanes 5 and 7 with lane 1), although CYLD and Optn levels in the nuclear fractions were only slightly increased (compare lanes 6 and 8 with lane 2). No cross-contaminations were found in the different cell fractions when analyzed for the presence of cytosolic and nuclear compartment specific markers (respectively NEMO and RNA Polymerase II B subunit, RBP1).

**Fig 4:**
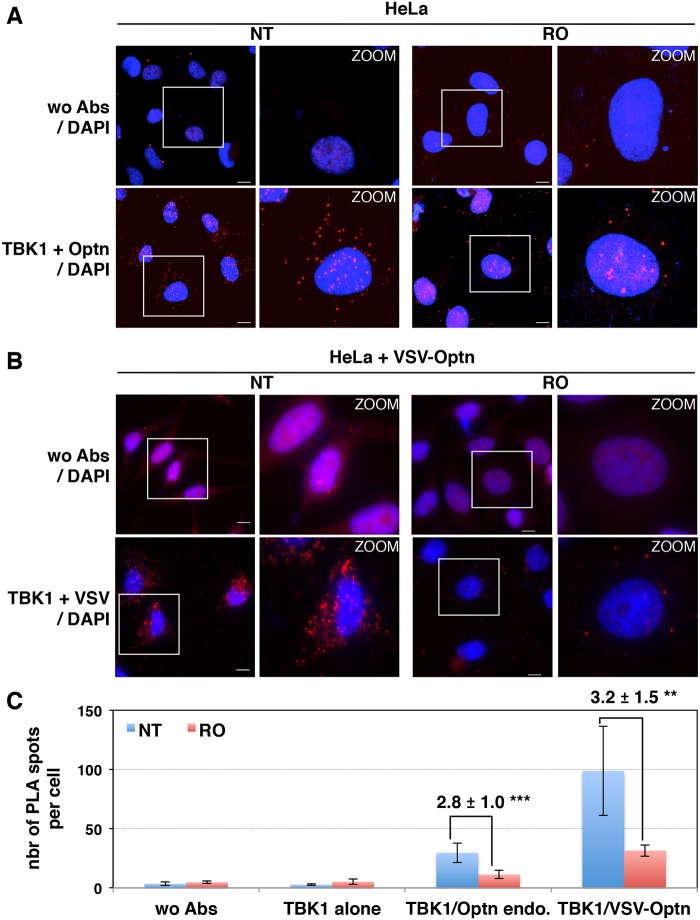
Interaction between TBK1 and Optn is prevented in G2/M synchronized cells. HeLa cells (A) and Optn-deficient HeLa cells expressing VSV-Optn (B), left untreated (NT) or synchronized in G2/M by RO-3306 (RO), were analyzed by *in situ* proximity ligation assays (PLA) using anti-TBK1 and anti-Optn (in A, lower panels) or anti-VSV (in B, lower panels) antibodies. Control PLA experiments were performed in the absence of antibodies (wo Abs, A and B, upper panels). Magnified views (x5 zoom factor) of the white square area are presented. Bars = 10 μm. (C) Quantification of the number of PLA spots per cell. In each condition, an average of 23 to 158 cells was imaged and 200 to 10.000 spots were counted using Imaris. Fold differences between PLA spots/cell observed with TBK1/Optn antibodies in HeLa cells (in A) or with TBK1/VSV antibodies in HeLa cells (in B) relative to the spots observed in the absence of antibodies (wo Abs) are indicated. p values < 0.01, *** p values < 0.001.

**Fig 5:**
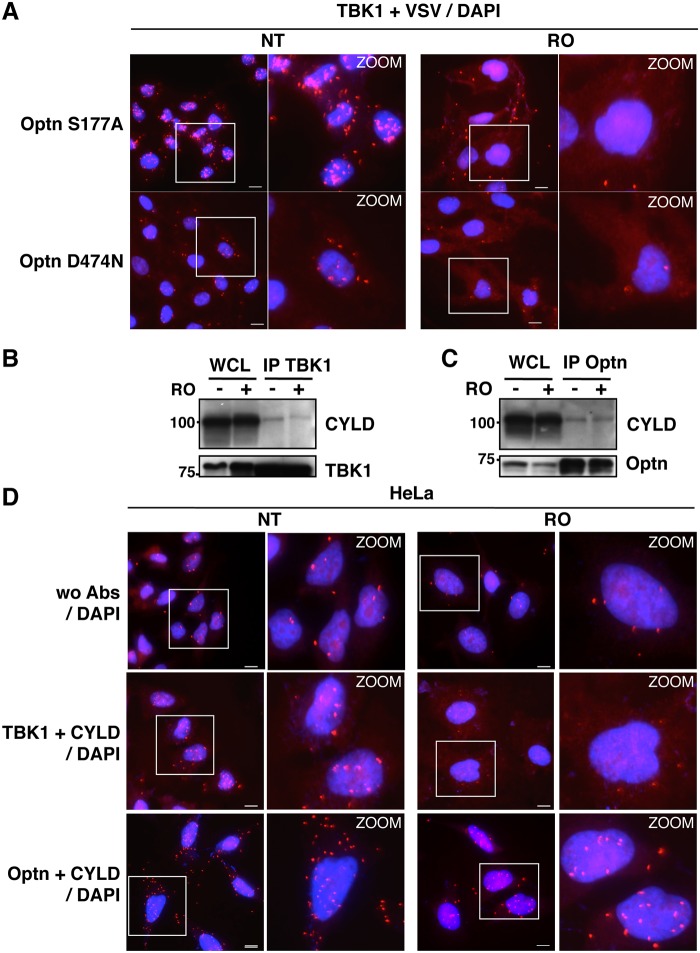
Characterization of the TBK1/CYLD- and Optn/CYLD-containing complex formation in G2/M synchronized cells. (A) Optn-deficient HeLa cells expressing VSV-Optn mutants (S177A or D474N), left untreated (NT) or synchronized in G2/M by RO-3306 (RO), were analyzed by *in situ* Proximity Ligation Assay (PLA) using anti-TBK1 and anti-VSV antibodies. Magnified views (x5 zoom factor) of the white square area are presented. Bars = 10 μm. (B-C) Whole cell lysate (WCL) from HeLa cells left untreated (-) or synchronized in G2/M by RO-3306 treatment (+) were immunoprecipited with anti-TBK1 antibodies and immunoblot with anti-CYLD and anti-TBK1 antibodies (A) or immunoprecipited with anti-Optn antibodies and immunoblot with anti-CYLD or anti-Optn antibodies (B). (D) HeLa cells left untreated (NT) or synchronized in G2/M by RO-3306 (RO) were analyzed by PLA in the absence (control wo Abs, upper panels) or in the presence of anti-TBK1 and anti-CYLD (middle panels) or in the presence of anti-Optn and anti-CYLD antibodies (lower panels). Magnified views (x5 zoom factor) of the white square area are presented. Bars = 10 μm.

We then hypothesized that the accumulation of Optn/CYLD to the nucleus during G2/M phase should disrupt the TBK1-Optn-CYLD complex formation. To visualize this complex that was hardly detectable by immunoprecipitation experiments of endogenous proteins, we used *in situ* Proximity Ligation Assays (PLA) that allows detection of interactions by generating a discrete and localized signal. Specificity of the antibodies used in PLA was confirmed by immunofluorescence using siRNA-mediated depletion of Optn, CYLD and TBK1 (S4H and S4I Fig). While only few non-specific punctate signals were observed in the absence of antibody or when the antibodies were used separately (Figs 4A, upper panels and S5A), a high number of dots were detected with both anti-TBK1 and anti-Optn in untreated parental or VSV-Optn expressing HeLa cells (Fig 4A and 4B, lower left panels). As a consequence of nuclear accumulation of Optn in G2/M synchronized cells, a dramatic reduction of the amounts of complexes formed between endogenous TBK1 and Optn (either endogenous or exogenous VSV-Optn) was observed after treatment of cells with RO-3306 (Fig 4A and 4B, lower right panels). Quantification of the PLA dots observed in the presence of anti-TBK1 and anti-Optn/VSV antibodies indicated a 3-fold decrease of the number of TBK1/Optn complexes in G2/M-synchronized cells compared to non-treated cells (Fig 4C). Since Optn inhibitory function in the IFN pathway requires its phosphorylation at S177 site and integrity of its ubiquitin-binding motif, we next assessed whether mutations of these motifs would interfere with Optn interaction with TBK1. Interestingly, expression of Optn S177A mutant did not apparently affect the formation, nor the RO-dependent dissociation of this complex, while this complex was hardly detectable when Optn D474N mutant was expressed (Fig 5A). Thus, unlike Optn phosphorylation at S177, ubiquitin-binding activity of Optn is critical for the TBK1/Optn interaction as previously shown [36]. As expected, formation of the TBK1/CYLD complex was also reduced during the G2/M phase, while CYLD-Optn interaction remained unaffected as shown by co-immunoprecipitation and *in situ* PLA experiments performed in RO-treated cells (Fig 5B, 5C and 5D, middle and lower panels, respectively).

### The TBK1/IFN pathway is enhanced during the G2/M transition

**Fig 6:**
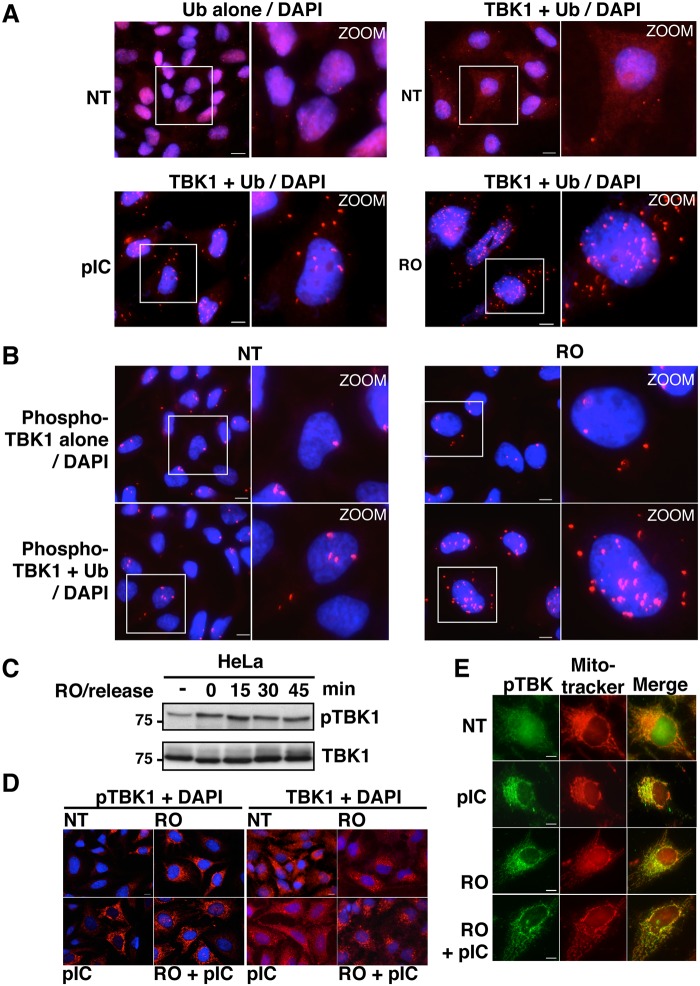
Activity and subcellular localization of TBK1 are altered in G2/M synchronized cells. (A) HeLa cells, left untreated (NT), stimulated by poly(I):poly(C) (pIC) or synchronized in G2/M by RO-3306 (RO), were analyzed by PLA using anti-ubiquitin antibodies alone (upper left panels) or in combination with anti-TBK1 antibodies. Magnified views (x5 zoom factor) of the white square area are presented. Bars = 10 μm. (B) HeLa cells left untreated (NT) or synchronized in G2/M by RO-3306 (RO) were analyzed by PLA using anti-pS172 TBK1 alone (upper panels) or in combination with anti-Ub antibodies. Magnified views (x5 zoom factor) of the white square area are presented. Bars = 10 μm. (C) Cell lysates from HeLa cells, treated with RO and released for the indicated periods of time (min), were immunoblotted with anti-pS172 TBK1 and anti-TBK1 antibodies. (D) Changes in localization of pS172-TBK1 (left panels) and total TBK1 pool (right panels) were monitored by immunofluorescence microscopy in HeLa cells untreated (NT) or synchronized by RO-3306 (RO) treatment and stimulated or not by poly(I):poly(C) (pIC). Bars = 10 μm. (E) Co-localization of pS172-TBK1 and mitochondria was performed by immunofluorescence using Mitotracker staining in HeLa cells untreated (NT) or synchronized by RO-3306 (RO) treatment and stimulated or not by poly(I):poly(C) (pIC). Bars = 10 μm.

Disruption of the interaction between TBK1 and CYLD should lead to higher ubiquitination levels of this kinase and consequently to its hyperactivation. To address this issue, we speculated that *in situ* PLA experiments using anti-TBK1 and anti-ubiquitin antibodies could allow the detection of cellular ubiquitinated TBK1. Indeed, analysis of poly(I:C)-stimulated cells by this approach revealed a significantly higher amount of PLA-specific dots compared to non-treated cells or to control experiments performed with a single antibody (Fig 6A). To ensure the specificity of these dots, we preincubated the fixed and permeabilized cells with recombinant deubiquitinase (viral ovarian tumor or vOTU) before PLA assay, a procedure that resulted in a complete loss of the PLA-specific signal obtained using anti-TBK1 and anti-ubiquitin antibodies, clearly indicating that PLA signal was related to ubiquitinateion of TBK1 or associated proteins (S5B Fig, right panels). As control, classical immunofluorescence microscopy confirmed that treatment of cells with vOTU reduced dramatically the ubiquitin cellular levels without affecting the detection of other proteins such as Optn (S5B Fig, left panels), suggesting that vOTU removed ubiquitin from almost all ubiquitinated proteins in cells. Interestingly, the number of PLA-specific dots observed using anti-TBK1 and anti-ubiquitin antibodies was increased in RO-treated cells compared to untreated cells (Fig 6A), strongly suggesting that disruption of the TBK1/CYLD interaction during the G2/M transition led to increased ubiquitination of TBK1 and therefore to its activation [40].

Accordingly, it was recently found that TBK1 is activated at mitosis in A549 cells and that loss of TBK1 impaired mitotic phosphorylation of the mitotic Polo-like kinase 1 (Plk1) in TBK1-sensitive lung cancer cells [47]. We observed a similar increase in TBK1 activity in HeLa cells arrested at the G2/M phase (Fig 6B and 6C). Thus, TBK1 activity (monitored using anti-pS172 TBK1 antibody) is enhanced in G2/M-synchronized cells and further increased after 15 minutes of RO-3306 release, when cells enter mitosis (Figs 6C and S6A). Interestingly, PLA experiments using anti-phosphorylated TBK1 and anti-ubiquitin antibodies showed higher number of dots in G2/M arrested-cells (Fig 6B) compared to non-treated cells (lower left panels) or to phospho-TBK1 antibodies alone (upper panels), further suggesting a connection between ubiquitination and phosphorylation of TBK1 during G2/M phase.

Since the transport of TBK1 between membranous organelles such as the ER, Golgi and mitochondria was shown to be required for optimal induction of innate immune responses [48,49], we next used immunofluorescence microscopy to determine the subcellular localization of TBK1 in G2/M arrested cells. Treatment with poly(I:C), RO-3306 or combination of both led to the detection of the phosphorylated forms of TBK1 (detected by the anti-pS172-TBK1 antibodies) in punctate structures, while only weak or no signals were detected in untreated cells (Fig 6D, left panels). These structures colocalized with a mitochondrial marker (mitotracker), but not with a Golgi apparatus resident protein, i.e. GM130 (Figs 6E and S6B). Of note, mitochondrial localization of active TBK1 has only been reported in response to infection by DNA viruses [50]. Unlike its phosphorylated form, TBK1 colocalized with GM130, but not with mitotracker in both untreated and stimulated cells (Figs 6D and S6C). These data clearly indicated that, as in the case of viral infection, synchronization of cells at the G2/M phase activates TBK1 and induces its recruitment to the mitochondrial compartment in proximity to the MAVS signalosome and to IRF3 [51].

**Fig 7:**
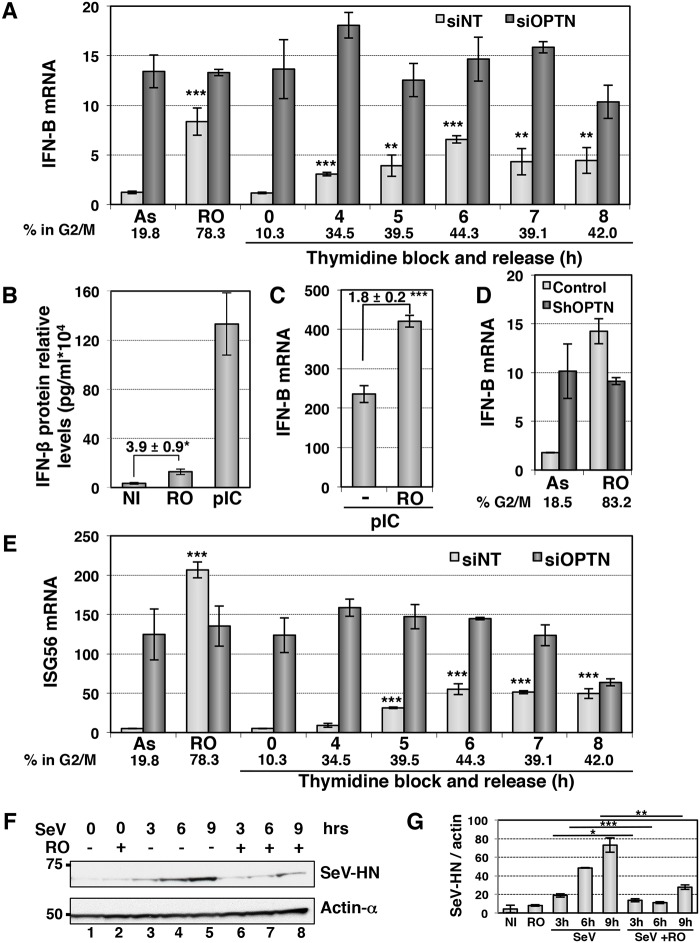
The IFN/ISG signaling pathway is induced in G2/M synchronized cells. (A) IFN-B mRNA levels were determined by RT-QPCR as described in Fig 1E, in HeLa cells transfected with non-target siRNAs (white bars) or with Optn-specific siRNAs (dark bars) and left unsynchronized (AS), blocked in G2/M phases by RO-336 treatment (RO) or blocked in G1/S transition by double thymidine block and release for the time indicated (in hours). Mean ± SD values of expression levels are presented. Paired t-test was used to determine the significance of the IFN-B level difference between asynchronous and synchronized cells. ** p values < 0.01, *** p values < 0.001. Average % of cells in G2/M determined by PI staining/FACS analysis for both siNT- and siOptn-transfected cells is shown. (B) IFN-β protein levels were determined by ELISA in the supernatants of HeLa cells left untreated (NI), synchronized in G2/M by RO-3306 (RO) or stimulated by poly(I:C) (pIC). Mean ± SD values of expression levels relative to 10^4^ cells are presented. Mean ± SD values of the induction folds corresponding to the ratio of the IFN-β protein level observed in RO-treated cells to that observed in control cells, is shown. * p values < 0.05. (C) IFN-B mRNA levels were determined by RT-QPCR in HeLa cells transfected with poly(I):(C) (pIC) and left unsynchronized (-), blocked in G2/M phases by RO-336 treatment (RO) as described in (A). Mean ± SD values of the induction folds corresponding to the ratio of the IFN-B expression level observed in RO-treated cells to that observed in control cells, is shown. *** p values < 0.001. (D) IFN-B mRNA levels were determined by RT-QPCR as described in (A) in control or stably Optn deficient HeLa cells transfected left unsynchronized (AS) or blocked in G2/M phases by RO-336 treatment (RO). The % of cells in G2/M determined in each condition by PI staining/FACS analysis is shown. (E) ISG56 mRNA levels were determined by RT-QPCR as described in (A), in HeLa cells transfected with non-target siRNAs or with Optn-specific siRNAs and left unsynchronized (AS), blocked in G2/M phases by RO-336 treatment (RO) or blocked in G1/S transition by double thymidine block and release for the time indicated (in hours). Mean ± SD values of expression levels, significance and average % of cells in G2/M are calculated as in (A). (F) Western blot analyses of extracts from asynchronous and RO-treated cells before and after different time (in hours) of SeV-infection using Hemagglutinin-Neuraminidase Protein of the Sendai virus (SeV-HN) and anti-actin-α antibodies. (G) Quantification of the signals obtained in (F) is presented as a ratio of SeV-HN protein levels versus actin levels. Paired t-test was used to determine the significance of the difference between the SeV-HN protein levels observed in asynchronous versus G2/M-treated cells after infection. * p values < 0.05, ** p values < 0.01, *** p values < 0.001.

Enhancement of TBK1 activity in G2/M synchronized cells should lead to increased IFN-B gene expression at this cell cycle phase independently of viral infection. Indeed, the basal expression level of IFN-B, that was barely detectable by RT-QPCR in asynchronous (AS) cells, was 7-fold increased when more than 50% of cells were blocked at G2/M phase following RO treatment (Figs 7A and S7A and S7B). Similarly, the enhancement of IFN-B expression observed following cell synchronization by thymidine block and release was correlated with the percentage of cells in G2/M phase. Accordingly, ELISA experiments indicated a 3- to 4-fold increase in the RO-induced IFN-β protein level, although this amount remains 10-fold lower than the IFN-β production following poly(I:C) stimulation (Fig 7B). This enhancement was linked to activation of the TBK1/IRF3 pathway as depletion of TBK1 resulted in strong inhibition of the RO-induced IFN-B gene expression, as for poly(I:C) stimulation (S7C and S7D Fig). The dsRNA-induced levels of IFN-B transcription was also increased in G2/M-arrested cells, since an almost 2-fold higher level of IFN-B gene expression was observed in RO-treated cells compared to unsynchronized cells following dsRNA stimulation (Fig 7C). Such a cell cycle-dependent regulation of the IFN-B gene expression was not observed in Optn-depleted cells (after siRNAs transfection), in which IFN-B transcription levels were high and unregulated regardless of the cell cycle stage (Fig 7A). Similar results were obtained in HeLa cells stably depleted for Optn and synchronized at the G2/M transition following RO treatment (Fig 7D).

### Protection against virus infection is enhanced during the G2/M transition

We next assessed the physiological role of this cell cycle-dependent regulation of the IFN production and found a similar cell cycle-dependent regulation of transcription of ISGs such as ISG56 (Fig 7E). Accordingly, we observed a 2- to 3- fold higher levels of luciferase activity when HL116 cells (expressing the luciferase gene controlled by the 6–16 ISG promoter) were incubated with supernatants from RO-treated compared to asynchronous HeLa cells, that were either left untreated, stimulated with dsRNA or infected by virus (S7E Fig). As expected, Optn depletion also led to higher 6-16-driven luciferase expression, which did not further increase upon RO treatment. Finally, we observed that replication of Sendai virus (monitored by Western blot experiments detecting the SeV-HN protein expression levels) was 2- to 4- fold decreased in HeLa cells synchronized at G2/M phase (Fig 7F and 7G). Increased protection against virus infection was also observed in antiviral cytopathic protection experiments (CPE), presented in S8A Fig, using supernatants from Sendai virus-infected (siNT SeV+RO) G2/M-synchronized Hela cells in comparison to asynchronous cells (siNT SeV). Additionally, depletion of Optn also significantly enhanced the protective effect of supernatants from SeV-infected cells against the Vesicular Stomatitis Virus infection (comparison of siNT SeV and siOptn SeV), indicating once again the negative regulatory role of Optn in the antiviral innate response.

Overall, these results suggest that Optn/CYLD dissociates from TBK1 following tehir accumulation in the nucleus during the G2/M transition, preventing the inhibition of TBK1 activity by the deubiquitinase activity of CYLD and thus promoting activation of the antiviral innate immune signaling pathway before cells enter into mitosis.

## Discussion

The production of type I IFN is a fundamental cellular response to protect an organism against viral invasion. Various viral components are recognized as pathogen-associated molecular patterns by specific receptors, among which RIG-like helicase receptor (RLR) family members were shown to play a critical role in the cytosolic sensing and host resistance against infection of a broad range of RNA viruses and some DNA viruses [3]. Given that RLRs are localized in the cytoplasm, the signaling pathway activated by these receptors should be tightly controlled to avoid autoactivation of the innate immune system in the absence of infection that could eventually lead to the development of autoimmune diseases [52,53]. Many studies have therefore focused on the mechanisms that regulate positively or negatively the RIG/MAVS/TBK/IRF pathway [3].

In the present study, we show that Optn acts as a negative regulator of the virus-induced IFN pathway by targeting the deubiquitinase CYLD to TBK1 in order to negatively regulate its activity. This finding first confirmed the previous observations, obtained by Mankouri et al. [36], describing Optn as an inhibitory effector of the innate immune system and further addressed the molecular mechanism of this negative regulatory function. Actually, we found that Optn targets TBK1 leading to downregulation of IRF3 phosphorylation levels and IFN-B gene expression. In agreement with other studies [14,35], our results indicate that TBK1-mediated phosphorylation of Optn occurs mainly on the S177 residue and is required for Optn inhibitory function, in addition to its ubiquitin-binding activity. We further showed that Optn targets the deubiquitinase CYLD to TBK1 in order to downregulate the antiviral innate immune pathway. This conclusion also originates from previous findings showing that CYLD is an interacting partner of Optn and a deubiquitinase for TBK1 [22,23]. We further demonstrate that Optn’s function in the innate immune pathway is related to its association with CYLD (but not with the deubiquitinase A20 or with a phosphatase complex) and that both Optn and CYLD are dependent on each other for their inhibitory effect on the IFN-B expression level.

Our results led us to propose a model for the regulation of TBK1 activity by Optn: In uninfected cells, the complex formed by TBK1, Optn and CYLD would allow constitutive deubiquitination and inhibition of TBK1, thereby limiting its activity in the absence of upstream signaling. Interestingly, formation of this complex relies not only on constitutive interactions between the interacting proteins, but also on modification-dependent interaction as shown by the loss of TBK1/Optn complex formation when an ubiquitin-binding deficient mutant of Optn is expressed. Following virus infection, activation of the RIG/MAVS pathway leads to TBK1 activation (presumably through phosphorylation by an upstream unknown kinase and ubiquitination by the E3 ligases MIB1/2 or Ndrp1) and to the disruption of its interaction with Optn and CYLD. At later times of infection, several TBK1-related mechanisms could be involved in the termination of IFN activation. First, dephosphorylated Optn could re-associate with CYLD to inhibit TBK1 activity. Second, active TBK1 may undergo K48-linked ubiquitination and degradation as previously hypothesized [22,54]. Moreover, as Optn is considered as an IFN-stimulated gene [29], an alternative mechanism could be provided by a negative feedback loop involving IFN-induced Optn expression. As Optn and NEMO share structural homologies, interact both with the deubiquitinase CYLD and are both involved as regulators of the RIG/TBK/IRF signaling pathway [16,27,45], a major issue will be to determine whether they interfere one with another through competitive interactions or coordinately act to regulate this pathway.

In this report, we have shown that Optn could exert a negative regulatory function on virus-induced IFN transcription in cells of fibroblastic origin in agreement with other studies [36,37]. In contrast, Optn was considered to play a positive role in LPS-mediated activation of TBK1 and IFN production in macrophages and dendritic cells [30,35] and it was found that TBK1 activity, IRF3 phosphorylation and IFN-β production were severely impaired in MEFs that express a polyubiquitin-binding defective mutant (D477N) of Optn [30,35]. These results apparently differ from those presented here, in particular the enhanced IFN-B expression in Optn-deficient cells reconstituted with Optn D474N mutant. These discrepancies could be explained by species differences. Indeed, unlike murine Optn D477N, human Optn D474N mutant did not interact with TBK1 (this study and [36]). It is also striking that the murine mutated form can apparently not be phosphorylated in response to LPS despite its interaction with TBK1 [35]. This hypothesis was further confirmed by comparing the effect of human D474N- and murine D477N-mutated versions of Optn in human HeLa and MEFs cells depleted from endogenous Optn by siRNA transfection (S8B and S8C Fig). Indeed, both human and murine wild-type Optn proteins negatively affected the pIC-stimulated expression of IFN-B, while only mutation of the human Optn in its UBD reverted its inhibitory effect. The similar or even higher inhibitory effect observed with the D477N-mutated mouse Optn compared to wt form that we observed in both human HeLa and MEF cells, clearly indicated that the human and murine Optn UBD-mutants are not functionally similar regarding the antiviral innate response, although they both are deficient for ubiquitin-binding activity [35]. It could be hypothesized that differential consequences of these mutations on CYLD interaction might explain their disparate effects on TBK1/IRF pathway. Yet, the inhibitory effect exerted by both human and murine Optn wt forms argues in favor of a negative regulatory role of Optn in the virus-induced IFN transcription when stimulated through the cytosolic RIG-dependent pathway.

We have previously shown that upon phosphorylation by Plk1 at S177, Optn recruits and delivers myosin phosphatase to the nucleus, thereby controlling Plk1 activity for proper mitotic progression [55]. Interestingly, the same Serine residue of Optn is phosphorylated by TBK1, and this phosphorylation is essential for Optn to function in the autophagy triggered by bacterial infection and in the antiviral innate immune responses ([14,38] and this study). In addition, both TBK1 and Plk1 interact with the upstream adaptor MAVS [56,57]. These findings raise the intriguing possibility that Optn may constitute a coordinating element of host defense and mitosis. Interplay between these two kinases has been complicated by the observation that TBK1 could target and activate Plk1 on Thr210 [47], but it will be interesting to determine whether phosphorylation by one of these kinases could promote or interfere with modification of Optn by the other and the functional consequences of this interplay. Our study revealed that the TBK1/IFN pathway is activated during the G2/M phase, as a consequence of Optn nuclear translocation and subsequent release of the CYLD-mediated inhibition of TBK1 kinase activity. Activation of TBK1 at mitosis was previously reported and further shown to promote phosphorylation of Plk1 and metadherin in TBK1-sensitive lung cancer cells to maintain their survival [47]. We similarly report that TBK1 is activated during the G2/M phase in the TBK1-insensitive HeLa cell line and provide a cell cycle-dependent mechanism for this activation. We now demonstrated that enhancement of TBK1 activity during the G2/M phase leads to increased IFN/ISG axis independently of viral infection. Constitutive secretion of IFN-β and other type I IFN at low amounts by many tissues of the body (close to the threshold level of detection) has already been reported and shown to maintain homeostasis and priming of cells to allow a rapid and robust innate and adaptive immune response to subsequent challenge (reviewed in [58]). In addition to immune functions, such as increase in myeloid cell function and regulation of macrophage/NK cell homeostasis, constitutive IFN-β secretion occurring in healthy animals is necessary for a diverse array of biological effects including maintenance of the hematopoietic stem cell niche and bone remodeling [59–61]. It is therefore tempting to speculate that IFN production during the G2/M phase accounts (at least in part) for the constitutive low amounts of IFN protein detected in tissues and for their role in preserving homeostasis, although part of this constitutive IFN-β transcription was suggested to rely on c-Jun/NF-κB pathways [62]. Alternatively, increased IFN secretion before mitosis could serve to protect cells against infection in this critical phase of the cell cycle where transcription is mostly shutdown, especially by maintaining the expression of IRF/STAT proteins and other signaling intermediates. Both activation of the Jak-Stat pathway during G2/M transition (as monitored by ISG expression analysis and HL116 cell assays), inhibition of Sendai virus protein expression and protective effects against VSV infection argue in favor of an antiviral function of the cell cycle-mediated IFN production.

Optn has been linked to various pathologies including primary and juvenile forms of Open-Angle Glaucoma and Amyotrophic Lateral Sclerosis (ALS) [32]. The familial primary open-angle glaucoma-associated E50K Optn mutant was reported to bind with higher efficiency to TBK1 [34] and the development of open angle glaucoma was observed in the course of interferon alpha therapy for chronic hepatitis B [63]. However, expression of E50K mutant in Optn-deficient cells restored the inhibitory effect of Optn on the innate immune response as efficiently as the wild type, indicating that association of this mutant with glaucoma does not seem to be linked to the role played by Optn in innate immunity. The effect of E50K Optn mutant in glaucoma has been rather linked to Optn turnover and to apoptosis in neuronal cells [64–66]. On the other hand, the ALS-associated mutants of Optn abrogate the inhibitory function of Optn on IRF3 activation in response to MDA5, or TRIF overexpression [37], suggesting a potential effect of these mutants on the TBK1/CYLD inhibitory mechanism. In the light of the close relationship between CYLD and apoptosis (reviewed in [41]), it could be speculated that the deregulation of the Optn/TBK1/CYLD axis could be involved in the neuronal cell death observed in Open Angle Glaucoma and familial ALS, independently of its effect on the antiviral immune pathway. This hypothesis have been very recently underscored by two studies identifying TBK1 as an ALS gene and showing that haploinsufficiency of TBK1 such as observed in the case of Optn-interaction mutant of TBK1, causes ALS and fronto-temporal dementia [67,68]. Finally, since Optn regulates positively the autophagic process to restrict bacterial growth [14] with same requirements as for its inhibitory antiviral function, it will be of interest to determine whether the relocalization of Optn/CYLD in the nucleus during the G2/M phase could also be involved in the regulation of the autophagic clearance of the cytosolic Salmonella.

## Materials and Methods

### Cells, infection and cell treatment

HeLa, HEK-293T, A549 (a kind gift from Eliane Meurs, Institut Pasteur, France) cells and Mouse Embryonic Fibroblasts (MEFs) were grown in DMEM supplemented with FBS (10%). HL116 cells (a kind gift from Sandra Pellegrini, Institut Pasteur, France [69]) were grown in DMEM supplemented with 10% FBS and HAT (Hypoxanthine: 20 μg/mL, Aminopterin: 0.2 μg/mL, Thymidine: 20 μg/mL). HeLa clones stably depleted for Optn expression were selected with 200 μg/ml of Geneticin (G418). HeLa cell lines expressing Optn wild type and mutants were selected with 1 μg/ml puromycin. Transient transfections of HeLa cells with siRNA and/or plasmids were performed using JetPrime (Ozyme/PolyPlus), Lipofectamine 2000 or Lipofectamine RNAiMAX (Invitrogen) following the manufacturer’s instructions (Eurogentec). Generation of Optn-deficient HeLa cell line (and their control with empty vector) and those reconstituted with wt or mutated Optn (E50K, S177A, F178R and D474N) was previously described [38]. For generation of HeLa cells reconstituted with L150/152P Optn mutant, pMSCV-puro vector (encoding the Optn construct) was transfected into Plat-A packaging cell line using FuGENE 6 (Roche) and retroviral-containing supernatants were harvested. High titers of recombinant viruses were obtained 48h after transfection and used to infect Optn-deficient cells. For Sendai virus infections (a kind gift from D. Garcin and E. Meurs), HeLa cells were incubated with the virus at m.o.i of 1 to 5 for 1h in serum free medium. Medium was then replaced by serum-complemented medium until harvesting. For stimulation of the intracellular pathways, HeLa cells were transfected with 2 μg/ml Poly(I)·Poly(C) (GE Healthcare/Amersham) or with 2 μg/ml of Poly(dA)·Poly(dT) (GE Healthcare/Amersham) using JetPrime (Ozyme/PolyPlus) according to manufacturer’s instructions. For TBK1 kinase inhibition, cells were pre-treated 4h with 1 μM of BX795 (Sigma-Aldrich) and induction was performed in the continuous presence of the drug.

A detailed list of antibodies, DNA constructs and chemicals is described in S1 Text.

### Cell extracts, immunoprecipitations and immunoblots

Lysis, immunoprecipitation and immunoblotting were performed as previously described [38]. For subcellular fractionations, cytoplasmic and nuclear extracts were obtained using the Subcellular Protein Fractionation Kit, according to the manufacturer’s instructions (Thermo Scientific). Cell lysis and purification of Ubiquitin conjugates was performed as described in Moretti et al. [70], at room temperature in denaturing conditions (8 M urea, 0.1 M NaH2PO4, 10 mM Tris-Hcl pH8, 1% Triton X-100 and 20 mM Imidazole). The chemiluminescence reaction was visualized and processed using myECL Imager system (Thermo Scientific). For relative quantification, densitometry analyses were performed using myImageAnalysis Software (Thermo Scientific). Band intensities in the linear range of the signal were measured using underexposed membranes. Two different concentrations of each sample were tested and samples with lower concentrations were used for analyses.

### Real-time RT-QPCR analysis

DNase I-treated total RNA was prepared using the NucleoSpin RNA L isolation kit (Macherey-Nagel). Reverse transcription reactions were primed with oligo(dT)_12-18_ (ThermoScientific) and performed with 2 μg of total RNA using the SuperScript reverse transcriptase (Life Technologies). QPCR assays were performed at least in triplicates using the Power Sybr Green Master Mix (Life Technologies) in CFX96 Real-Time PCR Detection System (Bio-Rad). Primer sets did not generate PCR products in the absence of reverse transcription. Primer pairs used to determine the expression levels of human IFN-B, ISG15, ISG56, Viperin, Optn, IκBα, 18S and murine IFN-B, Optn and GAPDH genes are presented in S1 Table. For determination of the constitutive expression levels of IFN-B and ISG56 genes, 4 μg of total RNA were used in reverse transcription reactions. The amplification product was identified by DNA sequencing and checked by the analysis of the melting curve in each assay. Relative expression levels are presented as mRNA copies relative to 18S mRNA copies. Standard deviation values indicated in the histograms derived from at least two independent experiments performed in triplicate. Two-tailed paired Student’s t-tests were used for generating p-values to determine statistical significance of virus-induced gene expression. In all analyses, the threshold for statistical significance was p<0.05.

### Immunofluorescence and *in situ* Proximity Ligation Assay

For immunofluorescence experiments, fixation, permeabilization and image acquisition were performed as previously described [38]. Staining of nuclei was performed with DAPI (Sigma) for 5 min. MitoTracker probe (Invitrogen) was added at 200 nM for 30 min at 37°C before fixation. *In situ* PLA experiments were performed according to the manufacturer’s instructions (Sigma-Aldrich/Olink Bioscience). PLA hybridization signals were observed using a fluorescent microscope (Zeiss Axio Imager Z1, using the Zeiss ApoTome system and the Zeiss Axiovision 4.8 software). For deubiquitinase treatment, fixed/permeabilized cells were incubated for 90 min at 37°C in the presence of 30 μM of the recombinant vOTU deubiquitinase (OTU domain from Crimean Congo hemorrhagic fever virus L1 protein, [71]). For quantification of PLA experiments, randomly selected fields were imaged on a LSCM (LSM700 from Zeiss, Jena, Germany) with a 63 × oil objective lens. Imaris (Bitplane, Zurich, Switzerland) was used to count the number of spots per cell. In each condition, an average of 23 to 158 cells was imaged and 200 to 10 000 spots were counted.

### Treatment with chemicals for cell cycle analyses and autophagy

For double Thymidine block and release, cells were first incubated with 2.5 mM Thymidine (Sigma) for 16h and then released for 8h. The Thymidine block was then repeated, and the second release was performed for the indicated period of time. For the G2/M border arrest, cells were treated with 9 mM RO-3306 (Calbiochem) during 20h and then released for the indicated period of time. Cells were washed with PBS, fixed in cold 70% ethanol, and resuspended in PBS containing 50 μg/mL propidium iodide (Sigma) and 200 μg/mL RNase A (Sigma). Analyses were performed with FACSCalibur or LSR Fortessa flow cytometer (BD bioscience) and FlowJo v10.0 software. To analyze the role of autophagy, HeLa cells were stimulated with poly(I:C) for 16h in the presence of autophagy inducers, such as 20 μM rapamycin or serum deprivation (starved in EBSS medium) and with autophagy inhibitors including 5 mM 3-Methyladenine (3-MA) and 200 nM bafilomycin A1.

### ELISA, IFN detection and antiviral-based Cytopathic effect (CPE) assay

The amounts of IFN-β protein in culture supernatants were determined using an ELISA kit (PBL Biomedical Laboratories, Piscataway, NJ) following the manufacturer’s supplied protocol. Optical densities taken at 450 nm for quantification were measured using the Thermo Scientific Multiskan EX plate reader. IFN secretion was quantified using the reporter cell line HL116 that carries the luciferase gene under the control of the IFN-inducible 6–16 promoter [69]. 5×10^4^ HL116 cells, plated 16 h prior the assay in 24-well plate, were incubated for 6 h with the desired culture supernatants or with human IFNα2a (500 U/ml) as control (PBL Biomedical Laboratories). Cells were then lysed (Luciferase Cell Culture Lysis, 5X Reagent, Promega) and luciferase activity measured using the Luciferase Assay Reagent (Promega) with Berthold LB960 luminometer apparatus. As treatment of cells with RO-3306 leads to cell proliferation arrest, luciferase activities were normalized relative to the concentration of proteins extracted from HeLa cells to compensate for the difference in cell proliferation with asynchronous cells. For antiviral cytopathic effect assay (CPE), 1.5×10^4^ A549 cells, seeded 16 h prior the assay in 96-well plate, were incubated for 20 h with the desired culture supernatants or with human IFNα2a (500 U/ml, PBL Biomedical Laboratories) as control. Cells are infected with various MOI (3x10^5^ pfu/ml for MOI of 1) of Vesicular Stomatitis Virus (VSV, provided by Eliette Bonnefoy, Université Paris Descartes, France) virus until the virus control showed > 90% CPE (approximately after 24h). Cells were washed once with PBS and subsequently fixed and stained (50 μl/well) with crystal violet/formaldehyde solution for 20 minutes at room temperature. Quantification was performed after addition of 2-Methoxyethanol by measuring optical densities at 550 nm using a TECAN Infinite F500 plate reader.

## Supporting Information

S1 Fig Optn negatively regulates the antiviral innate immune response. (A) Expression of the IFN-B gene measured by RT-QPCR in control, Optn-deficient and wild-type Optn reconstituted HeLa cells at 6h after Sendai virus (SeV) infection are presented as mRNA transcript levels related to the 18S mRNA (set at 100). Mean ± SD values of expression levels are shown. Mean ± SD values of induction folds (corresponding to the ratio of the IFN-B expression levels relative to that observed in HeLa cells) are shown. Paired t-test was used to determine the significance of the IFN-B level increase. ** p values < 0.01. (B) Expression of the ISG15, Viperin and IκBα genes measured by RT-QPCR in control, Optn-deficient and wild-type Optn reconstituted HeLa cells at 9h after Sendai virus (SeV) infection are presented as in (A). (C) IFN-B protein levels were determined by ELISA in the supernatants of control or Optn-depleted HeLa cells left untreated (NI) or stimulated by poly(I:C) (pIC). Mean ± SD values of expression levels relative to 10^4^ cells are presented. Mean ± SD values of the induction folds corresponding to the ratio of the IFN-β protein level observed in pIC-stimulated Optn-depleted HeLa cells to that observed in control HeLa cells, is shown. * p values < 0.05. (D) Dose-dependent stimulation of IFN-B expression determined by RT-QPCR as in (A) after transfection of Optn-deficient and wild-type Optn reconstituted HeLa cells with different concentrations (0.25, 0.5, 1, 2 and 4 μg/ml) of poly(I):poly(C) (pIC). (E) Expression of the IFN-B transcripts measured by RT-QPCR in HeLa cells transfected with increasing amounts of VSV-Optn expressing vector (0.125, 0.25, 0.5, 1 and 2 μg/ml) and stimulated by poly(I):poly(C) as described in (A). *Insert*: Total cell lysates from HeLa cells transfected and induced as described above, were immunoblotted with anti-VSV antibodies.
(TIF)

S2 Fig Optn inhibitory effect targets TBK1 activity. (A) Schematic representation of human Optn protein showing its structural domains and the localization of these domains relative to each other: coiled-coil regions (CC), Leucine zippers (LZ), zing fingers (ZF), putative helical domains (HLX), UBD domain, as well as a 166 aa insert region which is absent in NEMO and contains a putative Leucine zipper. Interfaces for interaction with polyubiquitin chains (Ub-binding) are indicated. Alignment of TBK1 phosphorylation consensus site with that of Optn and IRF3 proteins is shown. Potential phosphorylated Serine residues present in the [150–185] region of Optn are indicated. (B) Western blotting control of the experiments presented in Fig 1E. HeLa cells, Optn-deficient cells and deficient cells stably reconstituted with wt, D474N- or S177A-mutated forms of Optn were infected by Sendai virus, transfected with poly(I:C) (pIC) or with poly(dA:dT) (pdAdT). Whole cell Extracts from these cells were immunoblotted with anti-Optn antibodies. (C) IFN-B mRNA levels were determined by RT-QPCR in control HeLa cells, Optn-deficient cells and deficient cells reconstituted with wt, E50K- or LL/PP-mutated Optn that were infected by SeV for 6h. *Insert*: Total cell lysates from HeLa cells transfected and induced as described above, were immunoblotted with anti-VSV antibodies. (D) Western blotting control of the experiments presented in Fig 2A. Extracts from HeLa cells cotransfected with non-targeting (lanes 1–5) or Optn-specific (lanes 6–10) siRNAs together with empty vector (lanes 1 and 6) or plasmid expressing either constitutively active form of RIG (RIG-ΔN, lanes 2 and 7) and IRF3 (IRF3-5D, lanes 5 and 10) or native form of MAVS (lanes 3 and 8) and TBK1 (lanes 4 and 9) were immunoblotted with anti-Optn, anti-Myc, anti-TBK1 and anti-IRF3 antibodies. (E) Cell lysates from control and stable Optn-depleted cells transfected with poly(I):poly(C) (pIC) for the indicated periods of time (hours) were immunoblotted with anti-pS172 TBK1 and TBK1 antibodies. Quantification of the signals obtained was performed and presented as a ratio of phosphorylated levels versus total protein levels in the graph below.
(TIF)

S3 Fig Inhibitory effect of Optn on TBK1 is not related to Optn function in autophagy. (A) Whole cell lysate from HeLa cells non-transfected (lanes 1, 3, 4 and 6) or transfected with poly(I:C) (pIC, lanes 2 and 5) for 16h in the absence (lane 1) or presence of autophagy inducers: rapamycin (Rapa, 20 μM, lane 2) and nutrient deprivation (Nut. Dep., lanes 3 and 6) or the autophagy inhibitor bafilomycin A1 (baf 200 nM, lanes 4–6) were submitted to immunoblot with anti-TBK1 antibodies, anti-Optn and anti-LC3 antibodies or with anti-tubulin antibodies as loading control. The molecular weights (kDa) are represented on the left of immunoblot. (B) IFN-B mRNA levels were determined by RT-QPCR in control HeLa cells, Optn-deficient cells and deficient cells reconstituted with wt- or F178R-Optn that were transfected with poly(I:C) for 16h. (C) IFN-B (left panel) or Optn (right panel) mRNA levels were determined by RT-QPCR in HeLa cells transfected with non-targeting (siNT) or Optn-specific (siOptn) siRNAs and then stimulated by poly(I):poly(C) (pIC) in the absence or in the presence of rapamycin (Rapa, 20 μM). (D) IFN-B mRNA levels were determined by RT-QPCR in HeLa cells transfected with non-targeting (siNT) or Optn-specific (siOptn) siRNAs and then stimulated by poly(I):poly(C) (pIC) in the absence or in the presence of two autophagy inhibitors 3-Methyladenine (3-MA, 5 mM) or Bafilomycin A1 (Baf, 200 nM). (E) Western blotting control of experiment presented in S3D Fig. Whole cell lysate from HeLa cells transfected with non-targeting (siNT, lanes 1–6) or Optn-specific (siOptn, lanes 7–12) siRNAs and then stimulated by poly(I):poly(C) (pIC, lanes 4–6 and 10–12) in the absence or in the presence of 5 mM 3-Methyladenine (3-MA, lanes 2, 5, 8 and 11) or 200 nM Bafilomycin A1 (Baf, lanes 3, 6, 9 and 12) were submitted to immunoblot with anti-LC3, anti-phosphoS6 ribosomal protein, anti-Optn and anti-TBK1 antibodies. The molecular weights (kDa) are represented on the left of immunoblot.
(TIF)

S4 Fig Inhibitory effect of Optn on the IFN pathway requires CYLD. IFN-B (A) and IκBα (B) mRNA levels were determined by RT-QPCR in HeLa cells cotransfected with control (empty), CYLD- or A20-specific shRNA expressing plasmids and then stimulated by poly(I):poly(C) are presented as in S1A Fig. *Insert*: Total cell lysates from HeLa cells transfected and induced as described above, were immunoblotted with anti-CYLD and anti-A20 antibodies. (C) Western blotting control of the experiments presented in Fig 2C. HeLa cells were cotransfected with non-targeting (lane 1), Optn- (lanes 2 and 5) or CYLD-specific (lanes 3 and 7) siRNAs or/and with plasmids expressing CYLD (lanes 4 and 5) or Optn (lanes 6 and 7) and then stimulated by poly(I):poly(C). Whole cell extracts were prepared from these cells to perform immunoblotting experiments using anti-CYLD and anti-Optn antibodies. The molecular weights (kDa) are represented on the left of immunoblot. (D) Expression of the IFN-B transcripts measured by RT-QPCR in HeLa cells transfected with pcDNA3 plasmid, wild-type pCYLD or DUB-deficient H/N mutated CYLD (pCYLDm) expressing vectors and stimulated by poly(I):poly(C) as described in S1A Fig. Paired t-test was used to determine the significance of the IFN-B level difference in the absence and following CYLD overexpression. n.s. non significant, *** p values < 0.001. *Insert at bottom*: Total cell lysates from HeLa cells transfected with pcDNA3 plasmid (-), wild-type CYLD (wt) or DUB-deficient H/N mutated CYLD (mut.) and induced as described above, were immunoblotted with anti-CYLD antibodies. (E) Expression of the IFN-B transcripts measured by RT-QPCR in HeLa cells transfected with increasing amounts of VSV-Optn wt or VSV-Optn H486R expressing vectors (0.125, 0.25, 0.5, 1 and 2 μg/ml) and stimulated by poly(I):poly(C) as described in S1A Fig. *Insert above*: Total cell lysates from HeLa cells transfected and induced as described above, were immunoblotted with anti-CYLD antibodies. (F) Western blotting control of Fig 2D. Nickel-Sepharose-purified ubiquitinated products (Ni^2+^ beads) and whole cell lysates (WCL, 5% of the total lysates) were resolved on SDS-PAGE and analyzed using anti-Ubiquitin and anti-Optn antibodies. The molecular weights (kDa) are represented on the left of each immunoblot. (G) Western blotting control of Fig 2E. Whole cell lysates (WCL, 5% of the total lysates) were resolved on SDS-PAGE and analyzed using anti-CYLD antibodies. (H) Control of the experiments presented in Fig 3A. HeLa cells transfected with non-targeting (siNT), Optn- (siOptn) or CYLD-specific (siCYLD) siRNAs and treated or not with RO-3306 (RO) were analyzed by immunofluorescence using anti-CYLD or anti-Optn antibodies. Bars = 10 μm. (I) Control of the experiments presented in Fig 6D. HeLa cells transfected as indicated were analyzed by immunofluorescence using anti-Optn or anti-TBK1 antibodies. Bars = 10 μm.
(TIF)

S5 Fig Analyzes of the Optn/TBK1/CYLD complex formation by *in situ* Proximity Ligation Assay. (A) Control of the experiments presented in Fig 5D. HeLa cells left untreated (NT) or synchronized in G2/M by RO-3306 (RO), were analyzed by *in situ* Proximity Ligation Assay (PLA) using anti-TBK1, anti-Optn or anti-CYLD antibodies alone. Magnified views (x5 zoom factor) of the white square area are presented. Bars = 10 μm. (B) Control of the experiments presented in Fig 6A. Fixed and permeabilized HeLa cells were treated or not with deubiquitinase (DUB) as described in the Materials and Methods section and analyzed by immunofluorescence using anti-Optn or anti-ubiquitin (Ub) antibodies or by *in situ* Proximity Ligation Assay (PLA) using anti-TBK1 and anti-Ub antibodies. Magnified views (x5 zoom factor) of the white square area are presented. Bars = 10 μm.
(TIF)

S6 Fig Characterization of TBK1 activity and localization during the G2/M phase. (A) Control of the experiments presented in Fig 6C. HeLa cells left untreated (Asynchronous) or synchronized in G2/M by RO-3306 and released (RO-release) at different times indicated were submitted to FACS for cell cycle analysis. (B-C) Control of the experiments presented in Fig 6E. Co-localization of pS172-TBK1 (B) or TBK1 (C) and Golgi apparatus (GM130 marker, left panels) or mitochondria (Mitotracker, right panels) was performed by immunofluorescence in HeLa cells untreated (NT) or synchronized by RO-3306 (RO) treatment and stimulated or not by poly(I):poly(C) (pIC). Bars = 10 μm.
(TIF)

S7 Fig Changes in TBK1 localization during the G2/M phase lead to induction of the IFN/ISG signaling pathway. (A) IFN-B mRNA levels were determined by RT-QPCR in HeLa cells left unsynchronized (AS), blocked in G2/M phases by RO-336 treatment (RO) or blocked in G1/S transition by double thymidine block and release for the time indicated (in h). Mean ± SD values of expression levels are presented. Mean ± SD values of the induction folds, corresponding to the ratio of the IFN-B expression level observed in synchronized to that observed in asynchronized cells, is shown. ** p values < 0.01, *** p values < 0.001. The % of cells in G2/M determined in each condition by PI staining/FACS analysis is shown. (B) Control experiments of S7A Fig. HeLa cells left untreated (Asynchronous), synchronized in G2/M by RO-3306 or blocked in G1/S transition by double thymidine block and released (RO-release) at different times indicated were submitted to FACS for cell cycle analysis. (C) IFN-B mRNA levels were determined by RT-QPCR as described in (A) in HeLa cells transfected with non-targeting (siNT) or TBK1-specific (siTBK1) siRNAs left unsynchronized (AS) or blocked in G2/M phase by RO-336 treatment (RO) without (left graph) or followed by poly(I:C)-stimulation (right graph). Mean ± SD values of expression levels are presented. Mean ± SD values of the inhibitory effect of TBK1 siRNA is shown. ** p values < 0.01. (D) Western blotting control of experiments presented in (C) using anti-TBK1 and anti-Optn antibodies. (E) HeLa cells were transfected with non-targeting (siNT) or Optn-specific (siOptn) siRNAs, left unsynchronized (AS) or blocked in G2/M phase by RO-336 treatment (RO) followed by poly(I:C)-stimulation (pIC) or Sendai virus-infection (SeV, m.o.i of 5). Activity of type I IFN in the culture supernatants was measured using the HL116 reporter cell line after 16h of stimulation/infection. Luciferase activities were measured after 8h of contact with supernatants, normalized to the protein concentration extracted from HeLa cells. Data were obtained from three different luciferase assays performed with biologically independent duplicates of HeLa supernatants. Mean ± SD values of relative luciferase levels are presented. Mean ± SD values of the induction folds, corresponding to the ratio of the luciferase level observed in synchronized to that observed in asynchronized cells, is shown. * p values < 0.05, ** p values < 0.01.
(TIF)

S8 Fig Cytophatic protection experiments and functional analysis of ubiquitin-binding domain (UBD) mutants of the human and murine Optineurin. (A). Supernatants of HeLa cells, transfected and treated as indicated, were incubated with A549 cells for 20h that were further infected with different MOI of Vesicular Stomatitis Virus (VSV) for 24h. Living cells were stained by crystal violet that was then dissolved in 2-Methoxyethanol. Quantifications were done by measuring optical densities at 550 nm. Data were obtained from three different CPE assays performed with biologically independent duplicates of HeLa supernatants. Values are presented as the % of living cells relative to the signal obtained in non-infected conditions defined as 100%. Paired t-test was used to determine the significance of the difference between the protective effects observed in asynchronous versus G2/M-treated cells after infection. * p values < 0.05, ** p values < 0.01. (B and C). Human and mouse IFN-B (lefts panels) and Optn (right panels) mRNA levels were determined by RT-QPCR in HeLa cells (B) and MEFs (C) co-transfected with non-targeting (siNT) or Optn-specific (siOptn) siRNAs in the absence (-) or in the presence of either human or mouse Optn wt or UBD mutated forms (D474N-mutated human Optn or D477N-mutated mouse Optn, respectively) and then stimulated by poly(I):poly(C) (pIC). Mean ± SD values of inhibition folds (corresponding to the ratio of the IFN-B expression levels observed after Optn overexpression relative to that observed in the absence of Optn) are shown. Paired t-test was used to determine the significance of the IFN-B level inhibition. * p values < 0.05, ** p values < 0.01,*** p values < 0.001.
(TIF)

S1 Table List of primers for selected human and mouse genes used for Quantitative Real-Time PCR Analysis. (PDF)

S1 Text Supplementary materials and methods. Supplemental materials and methods and references for S1 Table.
(PDF)
