# Correction: Optineurin Regulates the Interferon Response in a Cell Cycle-Dependent Manner

**DOI:** 10.1371/journal.ppat.1004971

**Published:** 2015-06-11

**Authors:** Pierre Génin, Frédérique Cuvelier, Sandrine Lambin, Josina Côrte-Real Filipe, Elodie Autrusseau, Christine Laurent, Emmanuel Laplantine, Robert Weil

There is an error in the first sentence and fourth paragraph of the Introduction. The following sentence is incorrect:

Optineurin (tineurin (TE.DATA the MAVS-TRAOptn), also called NRP (NEMO-related protein) or FIP-2 (adenovirus E3-14.7K-interacting protein) exhibits 53% of sequence similarities with NEMO [28, 29].

The correct sentence is:

Optineurin (“optic neuropathy inducing” or Optn), also called NRP (NEMO-related protein) or FIP-2 (adenovirus E3-14.7K-interacting protein) is closely related to NEMO with 53% of sequence similarity [28,29].
