# Supplementary figures and images for: Optineurin Regulates the Interferon Response in a Cell Cycle-Dependent Manner
Source: PLoS Pathog. 2015 Apr 29;11(4):e1004877. doi: 10.1371/journal.ppat.1004877 (PMC4414543; doi:10.1371/journal.ppat.1004877)

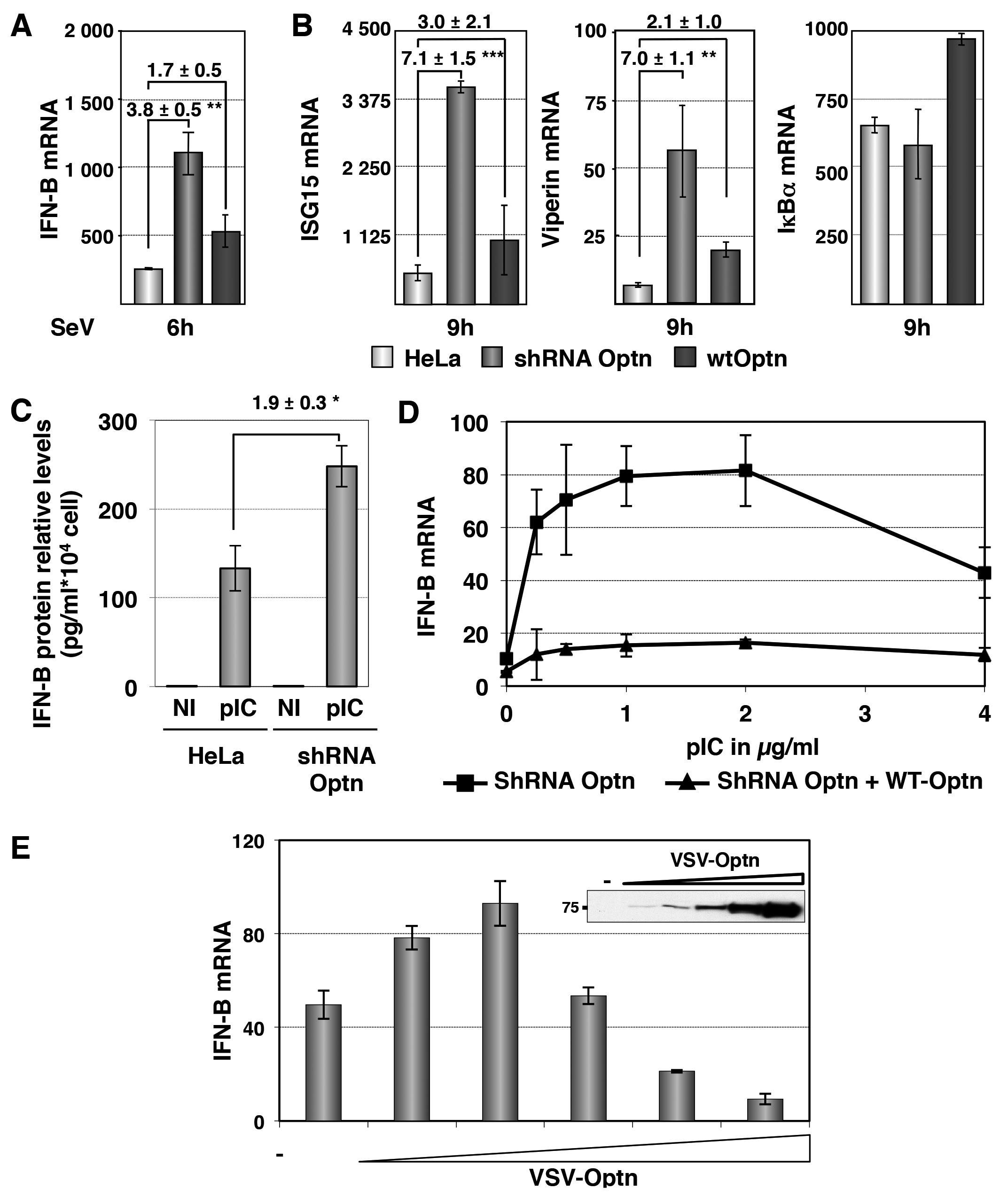

Supplement: S1 Fig — (A) Expression of the IFN-B gene measured by RT-QPCR in control, Optn-deficient and wild-type Optn reconstituted HeLa cells at 6h after Sendai virus (SeV) infection are presented as mRNA transcript levels related to the 18S mRNA (set at 100). Mean ± SD values of expression levels are shown. Mean ± SD values of induction folds (corresponding to the ratio of the IFN-B expression levels relative to that observed in HeLa cells) are shown. Paired t-test was used to determine the significance of the IFN-B level increase. ** p values < 0.01. (B) Expression of the ISG15, Viperin and IκBα genes measured by RT-QPCR in control, Optn-deficient and wild-type Optn reconstituted HeLa cells at 9h after Sendai virus (SeV) infection are presented as in (A). (C) IFN-B protein levels were determined by ELISA in the supernatants of control or Optn-depleted HeLa cells left untreated (NI) or stimulated by poly(I:C) (pIC). Mean ± SD values of expression levels relative to 104 cells are presented. Mean ± SD values of the induction folds corresponding to the ratio of the IFN-β protein level observed in pIC-stimulated Optn-depleted HeLa cells to that observed in control HeLa cells, is shown. * p values < 0.05. (D) Dose-dependent stimulation of IFN-B expression determined by RT-QPCR as in (A) after transfection of Optn-deficient and wild-type Optn reconstituted HeLa cells with different concentrations (0.25, 0.5, 1, 2 and 4 μg/ml) of poly(I):poly(C) (pIC). (E) Expression of the IFN-B transcripts measured by RT-QPCR in HeLa cells transfected with increasing amounts of VSV-Optn expressing vector (0.125, 0.25, 0.5, 1 and 2 μg/ml) and stimulated by poly(I):poly(C) as described in (A). Insert: Total cell lysates from HeLa cells transfected and induced as described above, were immunoblotted with anti-VSV antibodies. (TIF) [file ppat.1004877.s001.tif]

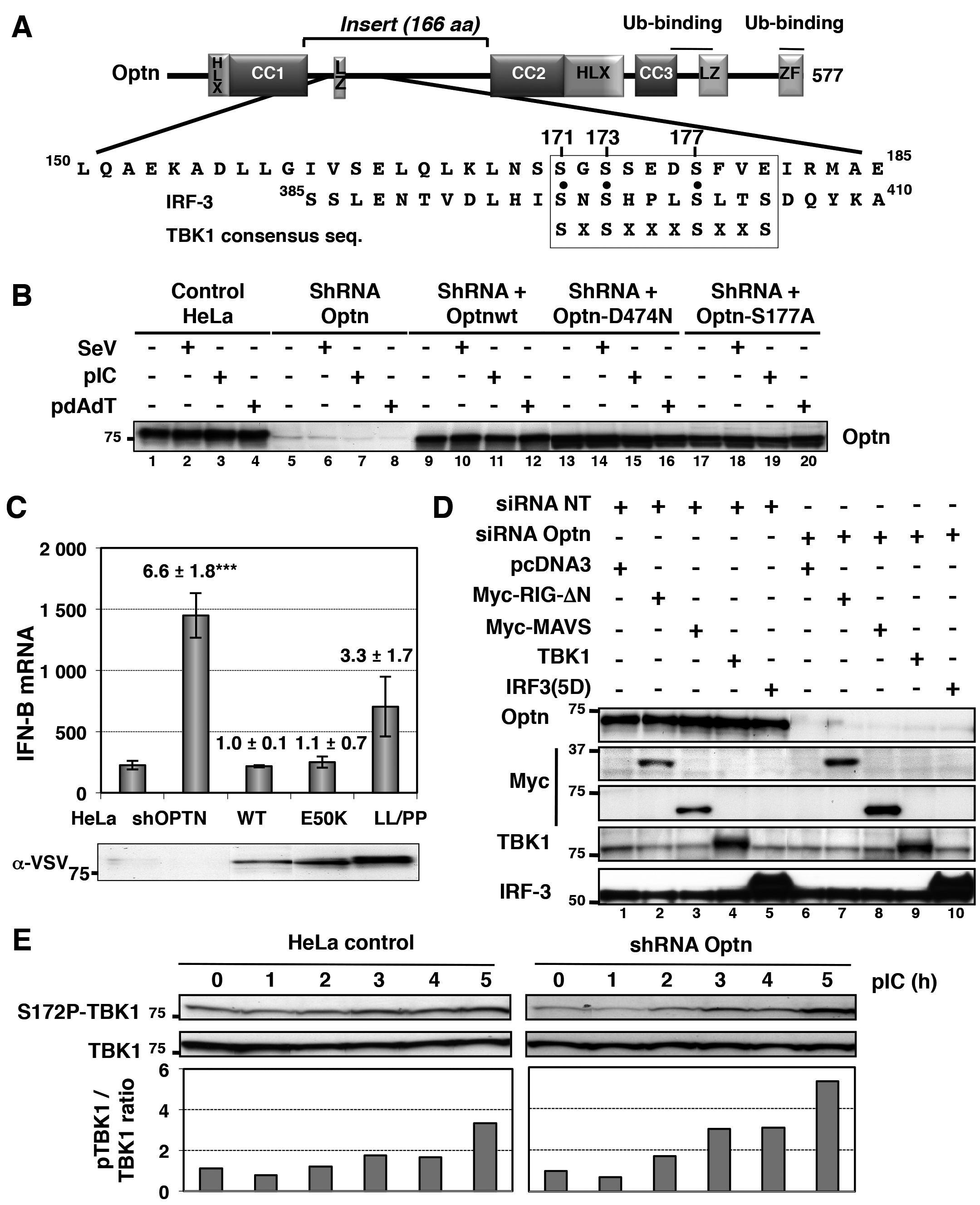

Supplement: S2 Fig — (A) Schematic representation of human Optn protein showing its structural domains and the localization of these domains relative to each other: coiled-coil regions (CC), Leucine zippers (LZ), zing fingers (ZF), putative helical domains (HLX), UBD domain, as well as a 166 aa insert region which is absent in NEMO and contains a putative Leucine zipper. Interfaces for interaction with polyubiquitin chains (Ub-binding) are indicated. Alignment of TBK1 phosphorylation consensus site with that of Optn and IRF3 proteins is shown. Potential phosphorylated Serine residues present in the [150–185] region of Optn are indicated. (B) Western blotting control of the experiments presented in Fig 1E. HeLa cells, Optn-deficient cells and deficient cells stably reconstituted with wt, D474N- or S177A-mutated forms of Optn were infected by Sendai virus, transfected with poly(I:C) (pIC) or with poly(dA:dT) (pdAdT). Whole cell Extracts from these cells were immunoblotted with anti-Optn antibodies. (C) IFN-B mRNA levels were determined by RT-QPCR in control HeLa cells, Optn-deficient cells and deficient cells reconstituted with wt, E50K- or LL/PP-mutated Optn that were infected by SeV for 6h. Insert: Total cell lysates from HeLa cells transfected and induced as described above, were immunoblotted with anti-VSV antibodies. (D) Western blotting control of the experiments presented in Fig 2A. Extracts from HeLa cells cotransfected with non-targeting (lanes 1–5) or Optn-specific (lanes 6–10) siRNAs together with empty vector (lanes 1 and 6) or plasmid expressing either constitutively active form of RIG (RIG-ΔN, lanes 2 and 7) and IRF3 (IRF3-5D, lanes 5 and 10) or native form of MAVS (lanes 3 and 8) and TBK1 (lanes 4 and 9) were immunoblotted with anti-Optn, anti-Myc, anti-TBK1 and anti-IRF3 antibodies. (E) Cell lysates from control and stable Optn-depleted cells transfected with poly(I):poly(C) (pIC) for the indicated periods of time (hours) were immunoblotted with anti-pS172 TBK1 and TBK1 an [file ppat.1004877.s002.tif]

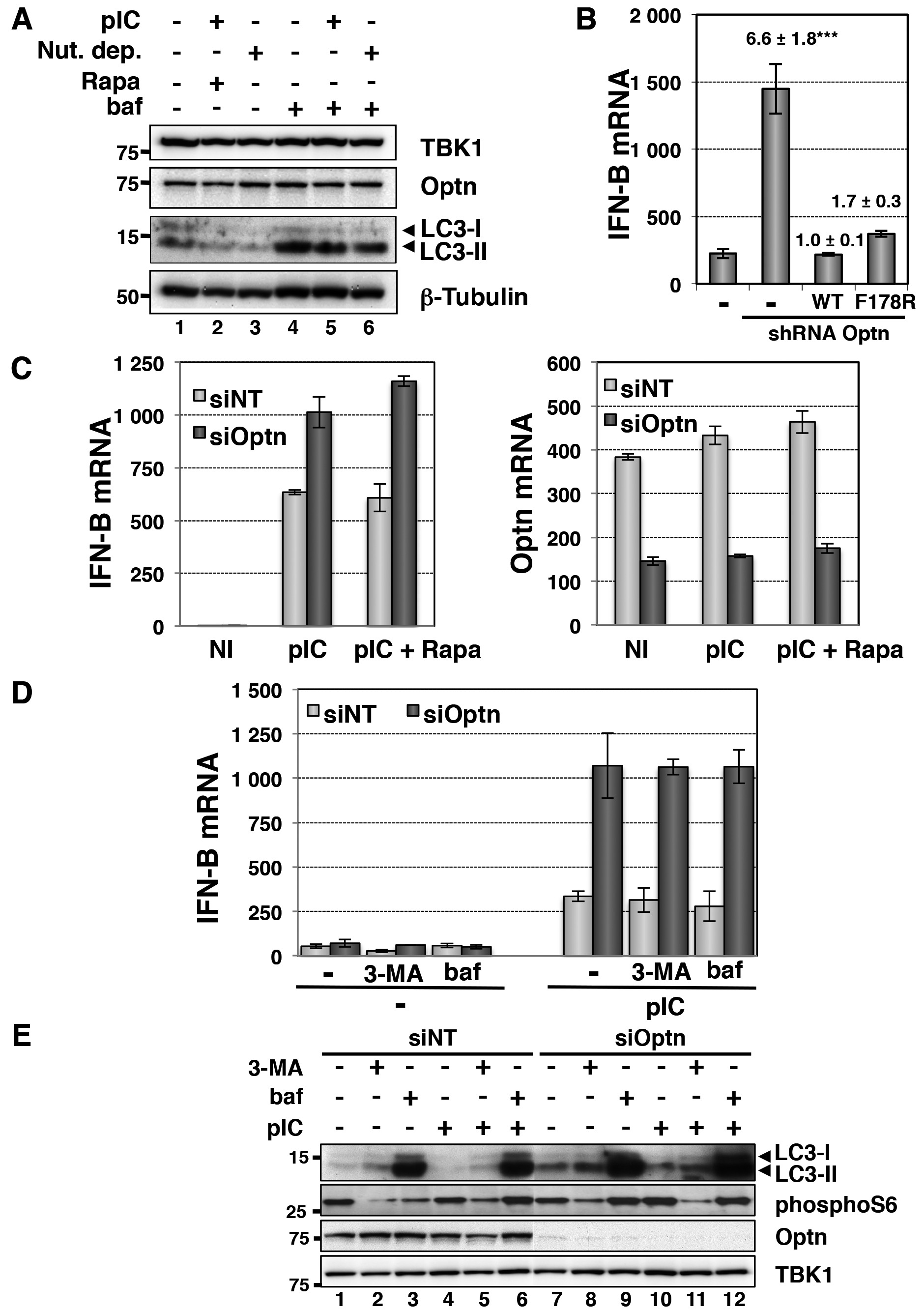

Supplement: S3 Fig — (A) Whole cell lysate from HeLa cells non-transfected (lanes 1, 3, 4 and 6) or transfected with poly(I:C) (pIC, lanes 2 and 5) for 16h in the absence (lane 1) or presence of autophagy inducers: rapamycin (Rapa, 20 μM, lane 2) and nutrient deprivation (Nut. Dep., lanes 3 and 6) or the autophagy inhibitor bafilomycin A1 (baf 200 nM, lanes 4–6) were submitted to immunoblot with anti-TBK1 antibodies, anti-Optn and anti-LC3 antibodies or with anti-tubulin antibodies as loading control. The molecular weights (kDa) are represented on the left of immunoblot. (B) IFN-B mRNA levels were determined by RT-QPCR in control HeLa cells, Optn-deficient cells and deficient cells reconstituted with wt- or F178R-Optn that were transfected with poly(I:C) for 16h. (C) IFN-B (left panel) or Optn (right panel) mRNA levels were determined by RT-QPCR in HeLa cells transfected with non-targeting (siNT) or Optn-specific (siOptn) siRNAs and then stimulated by poly(I):poly(C) (pIC) in the absence or in the presence of rapamycin (Rapa, 20 μM). (D) IFN-B mRNA levels were determined by RT-QPCR in HeLa cells transfected with non-targeting (siNT) or Optn-specific (siOptn) siRNAs and then stimulated by poly(I):poly(C) (pIC) in the absence or in the presence of two autophagy inhibitors 3-Methyladenine (3-MA, 5 mM) or Bafilomycin A1 (Baf, 200 nM). (E) Western blotting control of experiment presented in S3D Fig. Whole cell lysate from HeLa cells transfected with non-targeting (siNT, lanes 1–6) or Optn-specific (siOptn, lanes 7–12) siRNAs and then stimulated by poly(I):poly(C) (pIC, lanes 4–6 and 10–12) in the absence or in the presence of 5 mM 3-Methyladenine (3-MA, lanes 2, 5, 8 and 11) or 200 nM Bafilomycin A1 (Baf, lanes 3, 6, 9 and 12) were submitted to immunoblot with anti-LC3, anti-phosphoS6 ribosomal protein, anti-Optn and anti-TBK1 antibodies. The molecular weights (kDa) are represented on the left of immunoblot. (TIF) [file ppat.1004877.s003.tif]

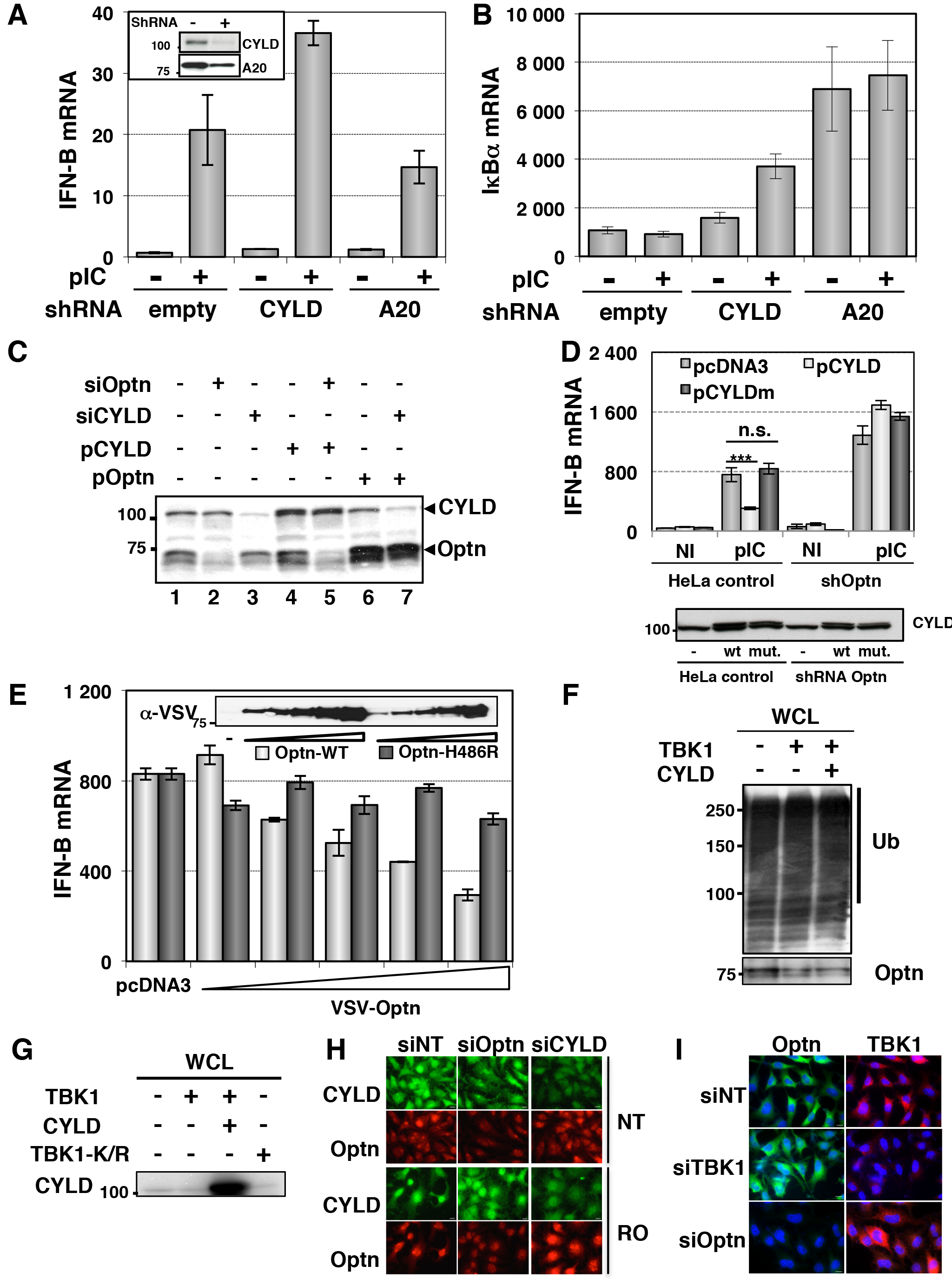

Supplement: S4 Fig — IFN-B (A) and IκBα (B) mRNA levels were determined by RT-QPCR in HeLa cells cotransfected with control (empty), CYLD- or A20-specific shRNA expressing plasmids and then stimulated by poly(I):poly(C) are presented as in S1A Fig. Insert: Total cell lysates from HeLa cells transfected and induced as described above, were immunoblotted with anti-CYLD and anti-A20 antibodies. (C) Western blotting control of the experiments presented in Fig 2C. HeLa cells were cotransfected with non-targeting (lane 1), Optn- (lanes 2 and 5) or CYLD-specific (lanes 3 and 7) siRNAs or/and with plasmids expressing CYLD (lanes 4 and 5) or Optn (lanes 6 and 7) and then stimulated by poly(I):poly(C). Whole cell extracts were prepared from these cells to perform immunoblotting experiments using anti-CYLD and anti-Optn antibodies. The molecular weights (kDa) are represented on the left of immunoblot. (D) Expression of the IFN-B transcripts measured by RT-QPCR in HeLa cells transfected with pcDNA3 plasmid, wild-type pCYLD or DUB-deficient H/N mutated CYLD (pCYLDm) expressing vectors and stimulated by poly(I):poly(C) as described in S1A Fig. Paired t-test was used to determine the significance of the IFN-B level difference in the absence and following CYLD overexpression. n.s. non significant, *** p values < 0.001. Insert at bottom: Total cell lysates from HeLa cells transfected with pcDNA3 plasmid (-), wild-type CYLD (wt) or DUB-deficient H/N mutated CYLD (mut.) and induced as described above, were immunoblotted with anti-CYLD antibodies. (E) Expression of the IFN-B transcripts measured by RT-QPCR in HeLa cells transfected with increasing amounts of VSV-Optn wt or VSV-Optn H486R expressing vectors (0.125, 0.25, 0.5, 1 and 2 μg/ml) and stimulated by poly(I):poly(C) as described in S1A Fig. Insert above: Total cell lysates from HeLa cells transfected and induced as described above, were immunoblotted with anti-CYLD antibodies. (F) Western blotting control of Fig 2D. Nickel-Sepharose-purified ubiquit [file ppat.1004877.s004.tif]

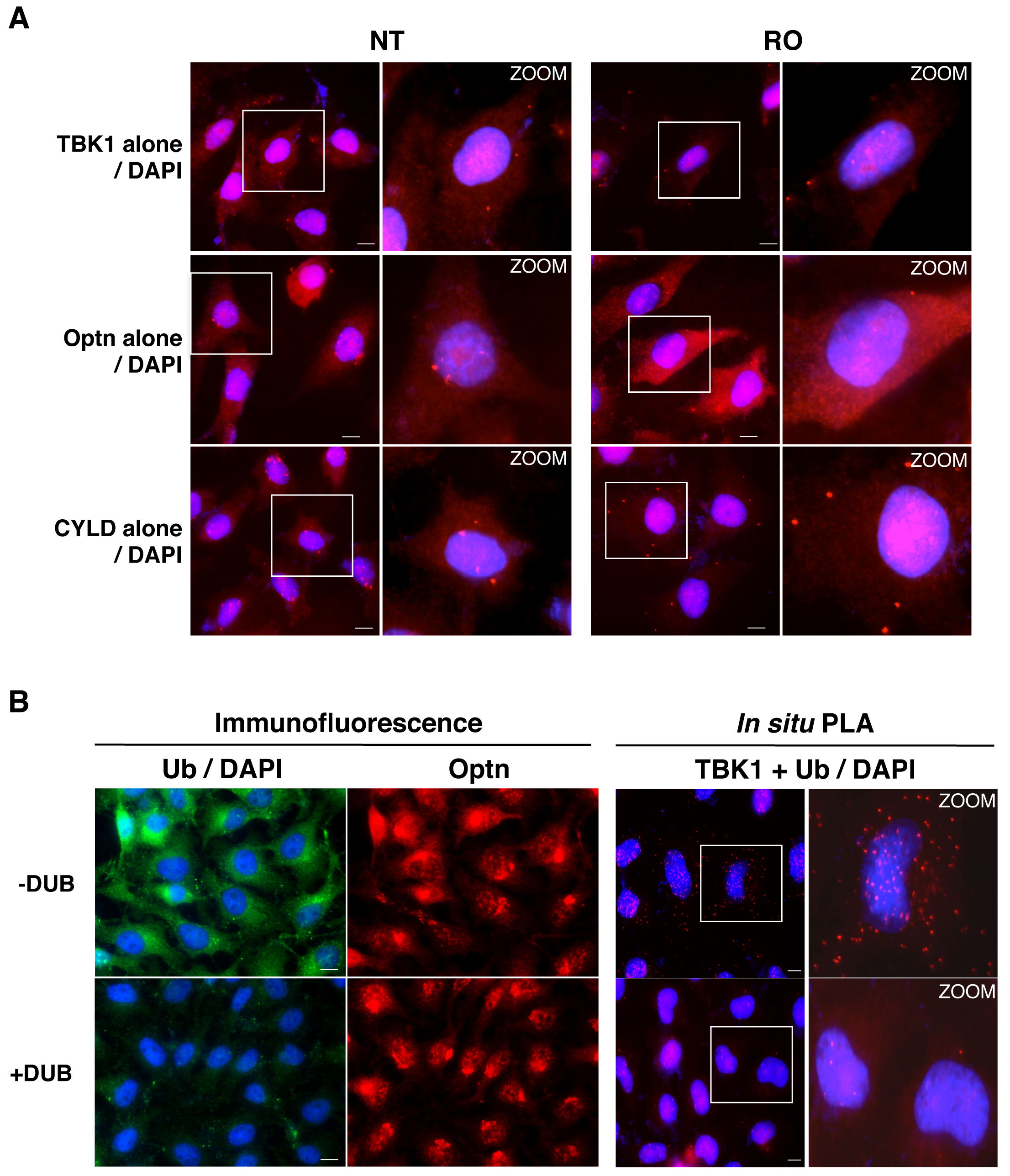

Supplement: S5 Fig — (A) Control of the experiments presented in Fig 5D. HeLa cells left untreated (NT) or synchronized in G2/M by RO-3306 (RO), were analyzed by in situ Proximity Ligation Assay (PLA) using anti-TBK1, anti-Optn or anti-CYLD antibodies alone. Magnified views (x5 zoom factor) of the white square area are presented. Bars = 10 μm. (B) Control of the experiments presented in Fig 6A. Fixed and permeabilized HeLa cells were treated or not with deubiquitinase (DUB) as described in the Materials and Methods section and analyzed by immunofluorescence using anti-Optn or anti-ubiquitin (Ub) antibodies or by in situ Proximity Ligation Assay (PLA) using anti-TBK1 and anti-Ub antibodies. Magnified views (x5 zoom factor) of the white square area are presented. Bars = 10 μm. (TIF) [file ppat.1004877.s005.tif]

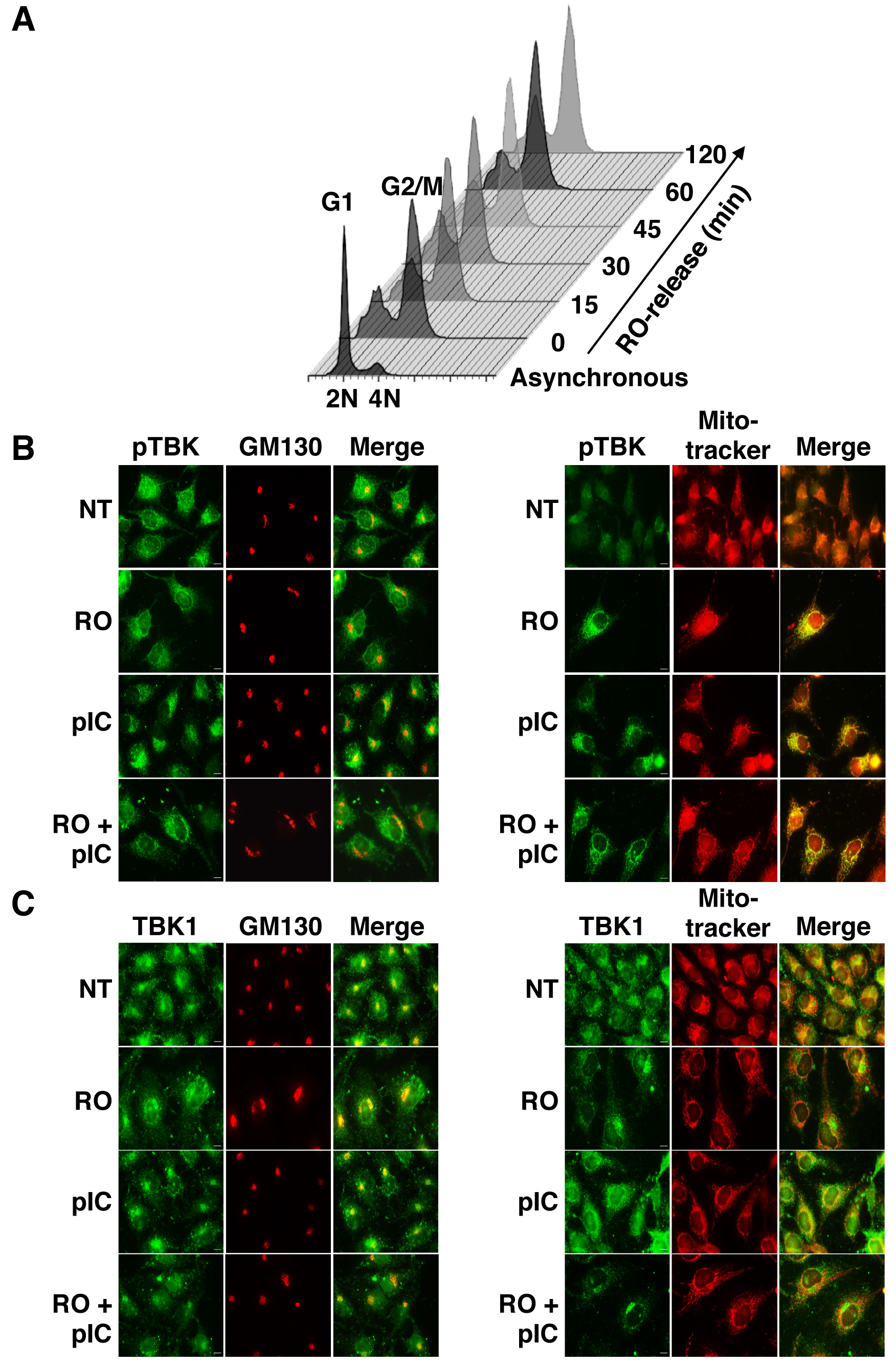

Supplement: S6 Fig — (A) Control of the experiments presented in Fig 6C. HeLa cells left untreated (Asynchronous) or synchronized in G2/M by RO-3306 and released (RO-release) at different times indicated were submitted to FACS for cell cycle analysis. (B-C) Control of the experiments presented in Fig 6E. Co-localization of pS172-TBK1 (B) or TBK1 (C) and Golgi apparatus (GM130 marker, left panels) or mitochondria (Mitotracker, right panels) was performed by immunofluorescence in HeLa cells untreated (NT) or synchronized by RO-3306 (RO) treatment and stimulated or not by poly(I):poly(C) (pIC). Bars = 10 μm. (TIF) [file ppat.1004877.s006.tif]

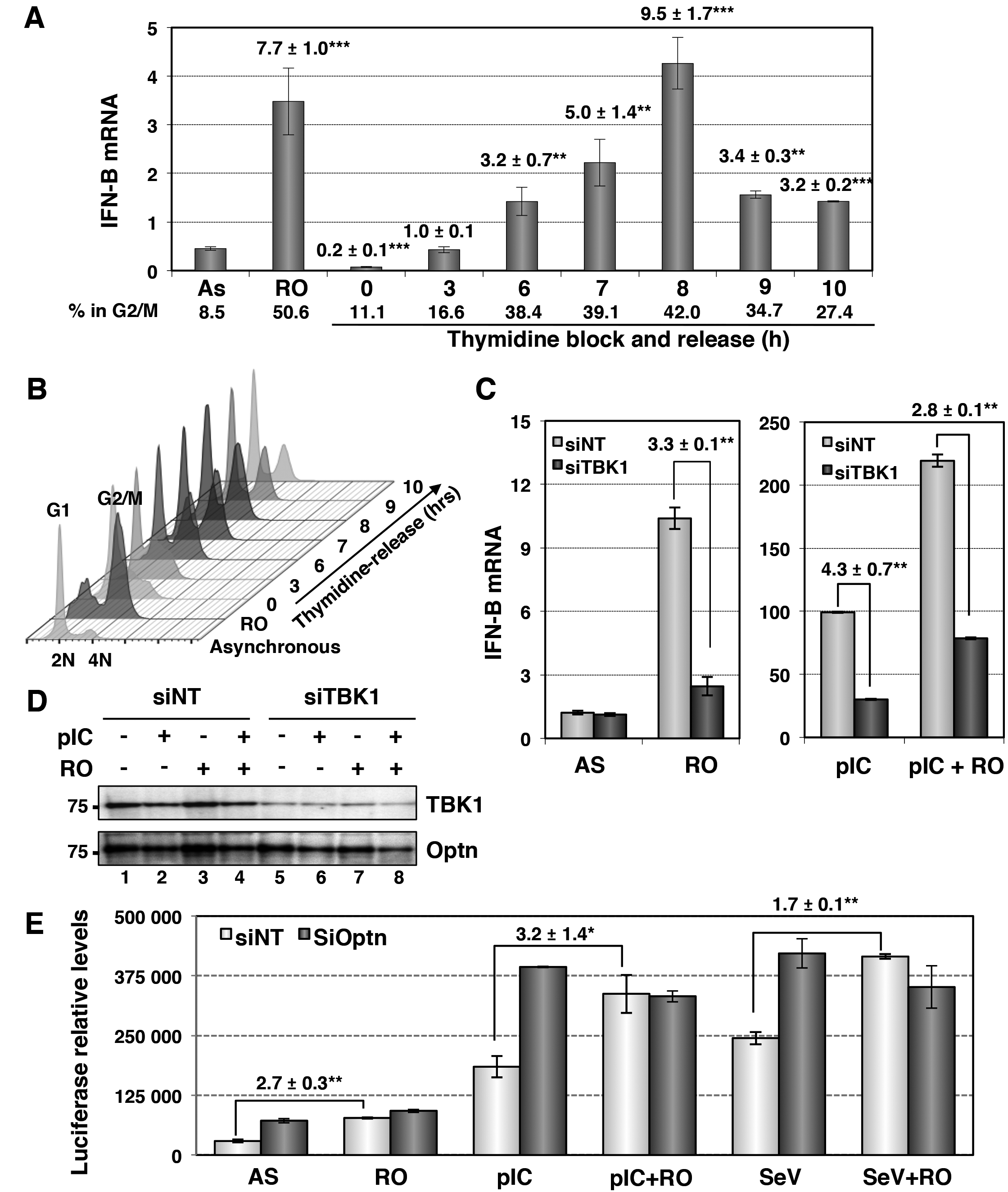

Supplement: S7 Fig — (A) IFN-B mRNA levels were determined by RT-QPCR in HeLa cells left unsynchronized (AS), blocked in G2/M phases by RO-336 treatment (RO) or blocked in G1/S transition by double thymidine block and release for the time indicated (in h). Mean ± SD values of expression levels are presented. Mean ± SD values of the induction folds, corresponding to the ratio of the IFN-B expression level observed in synchronized to that observed in asynchronized cells, is shown. ** p values < 0.01, *** p values < 0.001. The % of cells in G2/M determined in each condition by PI staining/FACS analysis is shown. (B) Control experiments of S7A Fig. HeLa cells left untreated (Asynchronous), synchronized in G2/M by RO-3306 or blocked in G1/S transition by double thymidine block and released (RO-release) at different times indicated were submitted to FACS for cell cycle analysis. (C) IFN-B mRNA levels were determined by RT-QPCR as described in (A) in HeLa cells transfected with non-targeting (siNT) or TBK1-specific (siTBK1) siRNAs left unsynchronized (AS) or blocked in G2/M phase by RO-336 treatment (RO) without (left graph) or followed by poly(I:C)-stimulation (right graph). Mean ± SD values of expression levels are presented. Mean ± SD values of the inhibitory effect of TBK1 siRNA is shown. ** p values < 0.01. (D) Western blotting control of experiments presented in (C) using anti-TBK1 and anti-Optn antibodies. (E) HeLa cells were transfected with non-targeting (siNT) or Optn-specific (siOptn) siRNAs, left unsynchronized (AS) or blocked in G2/M phase by RO-336 treatment (RO) followed by poly(I:C)-stimulation (pIC) or Sendai virus-infection (SeV, m.o.i of 5). Activity of type I IFN in the culture supernatants was measured using the HL116 reporter cell line after 16h of stimulation/infection. Luciferase activities were measured after 8h of contact with supernatants, normalized to the protein concentration extracted from HeLa cells. Data were obtained from three different luciferase assays perf [file ppat.1004877.s007.tif]

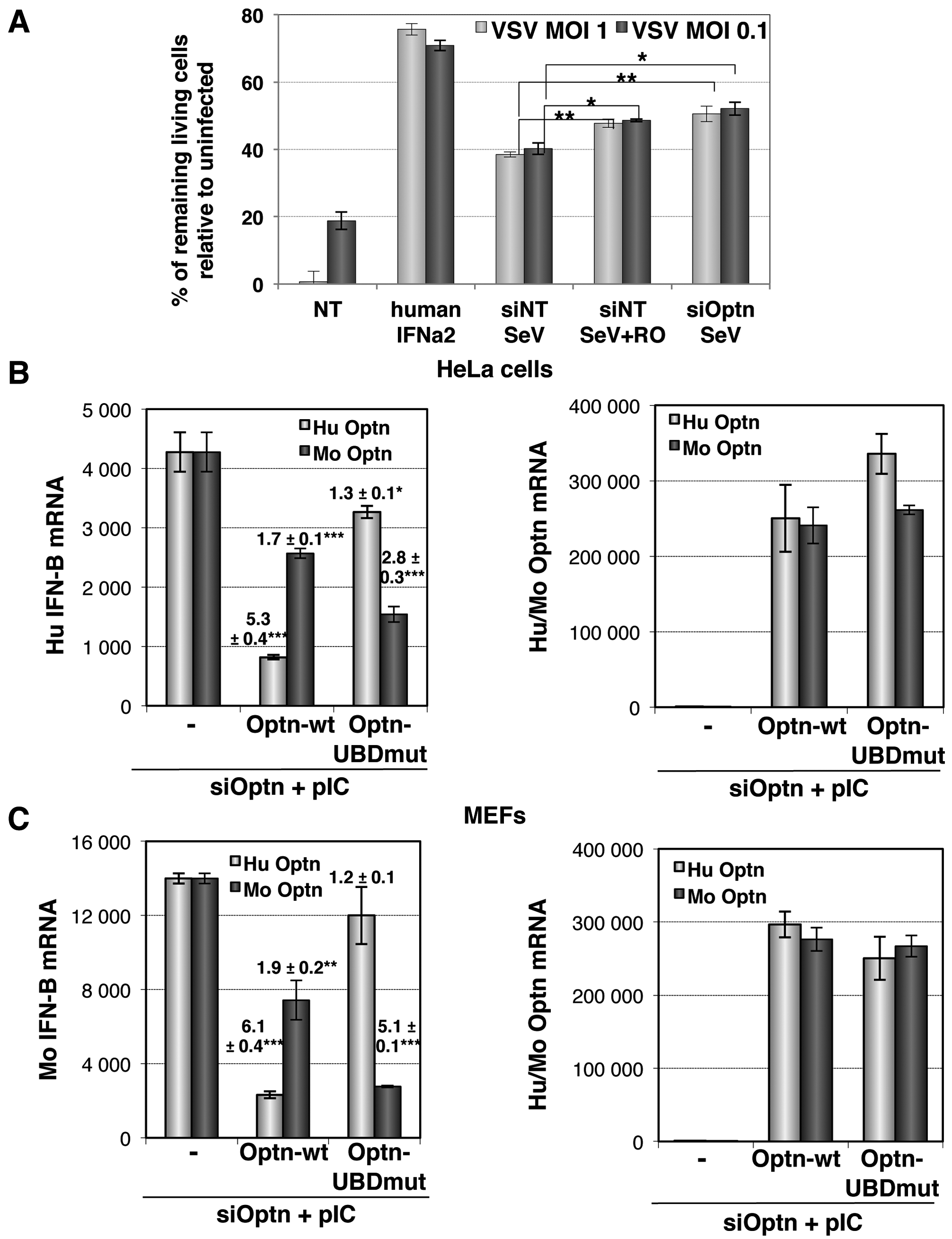

Supplement: S8 Fig — (A). Supernatants of HeLa cells, transfected and treated as indicated, were incubated with A549 cells for 20h that were further infected with different MOI of Vesicular Stomatitis Virus (VSV) for 24h. Living cells were stained by crystal violet that was then dissolved in 2-Methoxyethanol. Quantifications were done by measuring optical densities at 550 nm. Data were obtained from three different CPE assays performed with biologically independent duplicates of HeLa supernatants. Values are presented as the % of living cells relative to the signal obtained in non-infected conditions defined as 100%. Paired t-test was used to determine the significance of the difference between the protective effects observed in asynchronous versus G2/M-treated cells after infection. * p values < 0.05, ** p values < 0.01. (B and C). Human and mouse IFN-B (lefts panels) and Optn (right panels) mRNA levels were determined by RT-QPCR in HeLa cells (B) and MEFs (C) co-transfected with non-targeting (siNT) or Optn-specific (siOptn) siRNAs in the absence (-) or in the presence of either human or mouse Optn wt or UBD mutated forms (D474N-mutated human Optn or D477N-mutated mouse Optn, respectively) and then stimulated by poly(I):poly(C) (pIC). Mean ± SD values of inhibition folds (corresponding to the ratio of the IFN-B expression levels observed after Optn overexpression relative to that observed in the absence of Optn) are shown. Paired t-test was used to determine the significance of the IFN-B level inhibition. * p values < 0.05, ** p values < 0.01,*** p values < 0.001. (TIF) [file ppat.1004877.s008.tif]
