# Supplementary material for: Optineurin Regulates the Interferon Response in a Cell Cycle-Dependent Manner
Source: PLoS Pathog. 2015 Apr 29;11(4):e1004877. doi: 10.1371/journal.ppat.1004877 (PMC4414543; doi:10.1371/journal.ppat.1004877)
Supplement: S1 Table — (PDF) [file ppat.1004877.s009.pdf]

**Gene**	**Primer**	**Sequence (5' - 3')**	**product**	**Reference**
Human IFN-B	Forward<br>Reverse	GTC TCC TCC AAA TTG CTC TC<br>ACA GGA GCT TCT GAC ACT GA	114 bps	[11]
Human IκBα	Forward<br>Reverse	GAT CCG CCA GGT GAA GGG<br>GCA ATT TCT GGC TGG TTG G	102 bps	[12]
Human ISG15	Forward<br>Reverse	GTC CCT GAG CAG CTC CAT G<br>GTC CTG CAG CGC CAC ACC	120 bps	[13]
Human ISG56	Forward<br>Reverse	TGG AGT ACT ATG AGC GGG C<br>GGT GCC TAA GGA CCT TGT C	73 bps	[14]
Human Viperin	Forward<br>Reverse	TCA AAA GCT GAG GAG GTG GT<br>TTC CGT CAT GTC CTC TTC C	95 bps	[15]
Human Optn	Forward<br>Reverse	GAG AAG GCT CTG GCT TCC AA<br>GTC ATG GTT TCC AGG TCC TCT T	86 bps	-
Human GAPDH	Forward<br>Reverse	AGT CCA TGC CAT CAC TGC C<br>CCT GCT TCA CCA CCT TCT TG	263 bps	[11]
Human 18S	Forward<br>Reverse	AGG AAT TGA CGG AAG GGC AC<br>GGA CAT CTA AGG GCA TCA CA	318 bps	[12]
Murine IFN-B	Forward<br>Reverse	AAG AGT TAC ACT GCC TTT GCC ATC<br>CAC TGT CTG CTG GTG GAG TTC ATC	135 bps	[16]
Murine Optn	Forward<br>Reverse	GGA AGA GCG CCT GTT GTT TG<br>TCC AAG CTC TTC CTT CAG CC	97 bps	-
Murine GAPDH	Forward<br>Reverse	AAC TTT GGC ATT GTG GAA GG<br>GTG AGC TTC CCG TTC AGC TC	188 bps	[13]
