# Supplementary material for: Optineurin Regulates the Interferon Response in a Cell Cycle-Dependent Manner
Source: PLoS Pathog. 2015 Apr 29;11(4):e1004877. doi: 10.1371/journal.ppat.1004877 (PMC4414543; doi:10.1371/journal.ppat.1004877)
Supplement: S1 Text — Supplemental materials and methods and references for S1 Table. (PDF) [file ppat.1004877.s010.pdf]

Supplementary materials and methods.

Constructs and RNAi

The VSV-tagged-human-Optn and -Optn D474N plasmids were previously described [1]. Modified forms of Optn: VSV-tagged-Optn F178R, L150P/L157P (LL/PP), H486R and S162A-170A-171A-173A-174A-177A (S6A) were generated by site-directed mutagenesis with a PCR-based strategy. To construct plasmids pGST-Optn wt, S177A, S171A-173A and S171A-173A-177A, the fragment containing the corresponding coding region was PCR amplified and cloned into the pGEX 3x plasmid (Pharmacia). Flag-tagged TBK1 and IRF3-5D as well as myc-tagged RIG-DN and MAVS expressing plasmids were kindly provided by J. Hiscott. CYLD expressing plasmid has been previously described [2]. The pCMV-HA-mouse-Optineurin and pCMV-HA-mouse-Optineurin D477N constructs previously described [3], were provided by Philip Cohen (University of Dundee). The TBK1wt and TBK1-K30/401R mutant plasmids, previously described [4], were a kind gift from Michael J. Eck (Harvard Medical School) and David A. Barbie (Dana Farber Cancer Institute).

Human (5'-GGAGACUGUUGGAAGCGAAGUdTdT) and mouse (5'-AGAGAGUGUUGG AAGCGAAGUdTdT) Optn double-stranded siRNAs were purchased from Sigma-Aldrich. Non-target double-stranded siRNAs (smart pool) were purchased from Dharmacon. TBK1 and CYLD siRNA were previously described in Moretti et al. [5] and Kovalenko et al. [2], respectively. The pSuper plasmid expressing A20 and CYLD shRNAs were described in Moretti et al. [6] and Dirac et al. [7], respectively. The pSuper vector served as a non-silencing control. His_6-tagged and HA-tagged ubiquitin constructs were previously described [8].

Antibodies

The following antibodies were used: anti-VSV mAb (P5D4), anti-Myc mAb (9E10), anti-Flag

M2 mAb (F-1804, Sigma-Aldrich), anti-HA.11 mAb (MMS-101P, Covance), anti-GM130 mAb (610822, BD Biosciences), anti-RNA Polymerase B Subunit hRPB1 (PB-7C2, Euromedex), anti-NEMO rabbit polyclonal antibody (sc-8330, Santa Cruz Biotechnology), anti-β-tubulin mAb (T4026, Sigma), anti-actin mAb (clone AC40, Sigma), anti-IRF3 rabbit polyclonal (sc-9082, Santa Cruz biotechnology), anti-pS396 IRF3 rabbit monoclonal (4947, Cell Signaling), anti-TBK1 rabbit polyclonal (3013, Cell Signaling), anti-pS172 TBK1 rabbit monoclonal (5483, Cell Signaling), anti-CYLD mAb (sc-74435, Santa Cruz biotechnology), anti-A20 mAb (Imegenex, San Diego, CA), anti-ubiquitin mAb (clone FK2, 04-263, Millipore), anti-LC3 mAb (clone 5F10, Nanotools GmbH, München, Germany) and anti-phospho (Ser235/236)-S6 Ribosomal Protein rabbit mAb (4858, Cell Signaling) antibodies. Anti-Optn monoclonal mouse (D1.1), anti-Optn rabbit polyclonal antibodies as well as antibodies raised against Ser177-phosphorylated Optn (pS177) have been described previously [9]. Secondary antibodies for immunofluorescence were supplied by Molecular Probes (Alexa Fluor conjugates).

2D electrophoresis

Samples were treated with the ReadyPrep 2-D Cleanup Kit (BioRad) following manufacturer's instructions. The last pellet was resuspended in 2D sample buffer (urea 7M, thiourea 2M, Chaps 4%, Triton X100 2%, DTT 50 mM, ampholines 2%) and left 1h at room temperature. Protein concentrations were determined using RC DC Protein Assay (BioRad). A volume corresponding to 100 µg (or 170 µg) of protein was mixed with an appropriate volume of DeStreak rehydration solution (GE Healthcare) and applied to a 18-cm, narrow pH 4.5-5.5, immobilized pH gradient gel strip (IPG). The strips were then rehydrated overnight in a BioRad Protean isoelectric focusing (IEF) Cell and proteins were focused at 10 000 V for a total of 50 000 V-h. Strips were kept frozen (-80°C) until being processed for the second

dimension. Before the second dimension IPG strips were equilibrated at room temperature for 10 min in solution A (Tris 50 mM, urea 6M, SDS 2%, glycerol 20%) containing 2% DTT, and then 10 min in solution B (solution A without DTT but containing 2.5% iodoacetamide). Equilibrated IPG strips were then loaded onto a 7.5% polyacrylamide 20 x 23 cm slab vertical gel and proteins were separated according to their molecular weight. Our system allows the simultaneous run of 4 IPG strips thus ensuring optimum conditions of reproducibility between protein profiles. Standards for molecular masses were included in 2D-gels for calibration. They also served as landmarks to cut gels equally before transfer.

***In vitro* kinase assay**

As a source of holoenzyme, HEK 293T were transfected with a TBK1-expressing plasmid and lysed after 24h with 1X Chris buffer (0.5% NP-40, 50 mM Tris-HCl pH 8.0, 10% Glycerol, 0.1 mM EDTA, 200 mM NaCl) before immunoprecipitation. Production of fusion proteins and kinase assays were performed as described [10], by using the following kinase buffer: 20 mM Hepes, 10 mM $MgCl_2$, 50 mM NaCl, 2 mM EDTA, 5 mM DTT and 20 mM β-glycerophosphate (pH 7.5).

References

1. Journo C, Filipe J, About F, Chevalier SA, Afonso PV, et al. (2009) NRP/Optineurin Cooperates with TAX1BP1 to potentiate the activation of NF-kappaB by human T-lymphotropic virus type 1 tax protein. PLoS Pathogens 5: e1000521.
2. Kovalenko A, Chable-Bessia C, Cantarella G, Israel A, Wallach D, et al. (2003) The tumour suppressor CYLD negatively regulates NF-kappaB signalling by deubiquitination. Nature 424: 801-805.
3. Gleason CE, Ordureau A, Gourlay R, Arthur JS, Cohen P (2011) Polyubiquitin binding to optineurin is required for optimal activation of TANK-binding kinase 1 and production of interferon beta. J Biol Chem 286: 35663-35674.
4. Tu D, Zhu Z, Zhou AY, Yun CH, Lee KE, et al. (2013) Structure and ubiquitination-dependent activation of TANK-binding kinase 1. Cell Rep 3: 747-758.

5. Sharma S, tenOever BR, Grandvaux N, Zhou GP, Lin R, et al. (2003) Triggering the interferon antiviral response through an IKK-related pathway. Science 300: 1148-1151.
6. Moretti J, Chastagner P, Gastaldello S, Heuss SF, Dirac AM, et al. (2010) The translation initiation factor 3f (eIF3f) exhibits a deubiquitinase activity regulating Notch activation. PLoS Biology 8: e1000545.
7. Dirac AM, Nijman SM, Brummelkamp TR, Bernards R (2005) Functional annotation of deubiquitinating enzymes using RNA interference. Methods Enzymol 398: 554-567.
8. Moretti J, Chastagner P, Liang CC, Cohn MA, Israel A, et al. (2012) The ubiquitin-specific protease 12 (USP12) is a negative regulator of notch signaling acting on notch receptor trafficking toward degradation. J Biol Chem 287: 29429-29441.
9. Kachaner D, Filipe J, Laplantine E, Bauch A, Bennett KL, et al. (2012) Plk1-dependent phosphorylation of optineurin provides a negative feedback mechanism for mitotic progression. Mol Cell 45: 553-566.
10. Weil R, Schwamborn K, Alcover A, Bessia C, Di Bartolo V, et al. (2003) Induction of the NF-kappaB cascade by recruitment of the scaffold molecule NEMO to the T cell receptor. Immunity 18: 13-26.
11. Remoli ME, Giacomini E, Lutfalla G, Dondi E, Orefici G, et al. (2002) Selective expression of type I IFN genes in human dendritic cells infected with Mycobacterium tuberculosis. J Immunol 169: 366-374.
12. Yamamoto Y, Verma UN, Prajapati S, Kwak YT, Gaynor RB (2003) Histone H3 phosphorylation by IKK-alpha is critical for cytokine-induced gene expression. Nature 423: 655-659.
13. Ashley RL, Henkes LE, Bouma GJ, Pru JK, Hansen TR (2010) Deletion of the Isg15 gene results in up-regulation of decidual cell survival genes and down-regulation of adhesion genes: implication for regulation by IL-1beta. Endocrinology 151: 4527-4536.
14. Holzinger D, Jorns C, Stertz S, Boisson-Dupuis S, Thimme R, et al. (2007) Induction of MxA gene expression by influenza A virus requires type I or type III interferon signaling. J Virol 81: 7776-7785.
15. Rivieccio MA, Suh HS, Zhao Y, Zhao ML, Chin KC, et al. (2006) TLR3 ligation activates an antiviral response in human fetal astrocytes: a role for viperin/cig5. J Immunol 177: 4735-4741.
16. Roth-Cross JK, Martinez-Sobrido L, Scott EP, Garcia-Sastre A, Weiss SR (2007) Inhibition of the alpha/beta interferon response by mouse hepatitis virus at multiple levels. J Virol 81: 7189-7199.
